# A proposed evidence-guided algorithm for the adjustment and optimization of multi-function articulated ankle-foot orthoses in the clinical setting

**DOI:** 10.3389/fresc.2024.1353303

**Published:** 2024-07-24

**Authors:** Nicholas A. LeCursi, Beatrice M. Janka, Fan Gao, Michael S. Orendurff, Yufan He, Toshiki Kobayashi

**Affiliations:** ^1^ Becker Orthopedic, Inc., Troy, MI, United States; ^2^ Department of Kinesiology and Health Promotion, University of Kentucky, Lexington, KY, United States; ^3^ Oregon Biomechanics Institute, Ashland, OR, United States; ^4^ Deparment of Biomedical Engineering, The Hong Kong Polytechnic University, Kowloon, Hong Kong SAR, China

**Keywords:** ankle foot orthosis, gait, AFO, kinematics, kinetics, alignment, stiffness, resistance

## Abstract

Individuals with neuromuscular pathologies are often prescribed an ankle-foot orthosis (AFO) to improve their gait mechanics by decreasing pathological movements of the ankle and lower limb. AFOs can resist or assist excessive or absent muscular forces that lead to tripping, instability, and slow inefficient gait. However, selecting the appropriate AFO with mechanical characteristics, which limit pathological ankle motion in certain phases of the gait cycle while facilitating effective ankle movement during other phases, requires careful clinical decision-making. The aim of this study is to propose an explicit methodology for the adjustment of multi-function articulated AFOs in clinical settings. A secondary aim is to outline the evidence supporting this methodology and to identify gaps in the literature as potential areas for future research. An emerging class of AFO, the multi-function articulated AFO, offers features that permit more comprehensive, iterative, and reversible adjustments of AFO ankle alignment and resistance to ankle motion. However, no standard method exists for the application and optimization of these therapeutic devices in the clinical setting. Here we propose an evidence-guided methodology applicable to the adjustment of multi-function articulated AFOs in the clinical setting. Characteristic load–deflection curves are given to illustrate the idealized yet complex resistance-angle behavior of multi-function articulated AFOs. Research is cited to demonstrate how these mechanical characteristics can help mitigate specific pathologic ankle and knee kinematics and kinetics. Evidence is presented to support the effects of systematic adjustment of high resistance, alignable, articulated AFOs to address many typical pathomechanical patterns observed in individuals with neuromuscular disorders. The published evidence supporting most decision points of the algorithm is presented with identified gaps in the evidence. In addition, two hypothetical case examples are given to illustrate the application of the method in optimizing multi-function articulated AFOs for treating specific gait pathomechanics. This method is proposed as an evidence-guided systematic approach for the adjustment of multi-function articulated AFOs. It utilizes observed gait deviations mapped to specific changes in AFO alignment and resistance settings as a clinical tool in orthotic treatment for individuals with complex neuromuscular gait disorders.

## 1 Introduction

**Figure 1:**
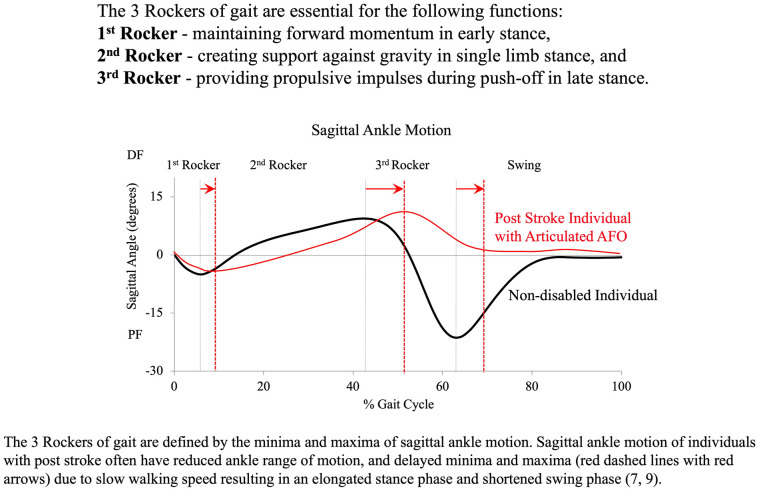
The three rockers of gait are defined by initial contact to first peak plantarflexion (First rocker: heel rocker), peak plantarflexion to peak dorsiflexion (Second rocker: ankle rocker), and peak dorsiflexion to second peak plantarflexion (Third rocker: forefoot rocker) (7, 9).

Ankle-foot orthoses (AFOs) are common assistive devices used to treat pathologic gait and help facilitate functional gait by improving ankle and knee motion in patients with neuromotor pathologies. In healthy individuals, efficient walking involves muscle activations to control the motion of the ankle and other joints to initiate or resist motion across various phases of the gait cycle (1–5). The ankle must perform a complex series of tasks during walking (6, 7) and the function of the ankle during gait may be described by dividing the gait cycle into foot-centric phases known as the “three rockers of gait” (1, 7, 8) (Figure 1).

For individuals with compromised neuromusculoskeletal systems, disrupted motion and forces acting at the ankle result in pathologic deviations that are primarily observed in the three rockers of gait, but can also include pathological kinematics and kinetics at the knee and hip. Pathologic ankle biomechanics can be positively influenced by an AFO that resists/assists ankle motion to compensate for impaired muscle function (10–15). Research demonstrates that an AFO can assist the ankle in improving stability and enhancing walking competence, efficiency, and mobility.

The primary indication for AFO prescription is excessive plantarflexion (PF) in swing phase for individuals with foot drop. This can lead to an increased risk of the patient tripping (16, 17). A secondary but related indication is toe-heel or flat foot gait at initial contact. This pathologic gait pattern severely disrupts the forward momentum of the body during ambulation (18). The position of the foot at initial contact, maximum plantarflexion in early stance, maximum ankle dorsiflexion (DF) during mid-stance, ankle push-off during terminal stance, and foot clearance in swing may all benefit from AFOs. Studies indicate that AFOs can improve joint kinematics and kinetics (19–23), walking speed (24), standing stability (24, 25), and energy efficiency (26, 27), leading to improved patient mobility and safety.

However, adjusting the mechanical properties of an AFO in the clinical setting to fully maximize these benefits for the patient is a complex task. This study proposes an evidence-guided algorithm for the adjustment of articulated AFO mechanical characteristics to remediate specific pathologic gait deviations and improve ankle and knee kinematics and kinetics throughout the gait cycle. This work aims to assist clinicians in establishing a more consistent evidence-guided clinical methodology for the adjustment of articulated AFOs. It is anticipated that this evidence-guided methodology may establish a foundation for future research into the method itself, potentially leading to further published evidence on the efficacy of this and other lower limb orthotic interventions.

There is a broad compendium of literature comparing the effectiveness of non-articulated and articulated AFOs in the treatment of gait deficits. Non-articulated AFOs are typically of the solid ankle, posterior leaf spring (PLS), or strut type. Articulated AFOs are typically known as hinged AFOs (24–26, 28) and frequently employ metal springs to resist/assist ankle motion. Researchers have investigated the influence of these AFO types when treating pathologic gait deviations, and compared different AFO designs for different patient populations (19, 28–30). More recently, systematic reviews have been conducted (24–26, 28) to compare gait in individuals post-stroke with and without the use of an AFO, regardless of the mechanical characteristics of the AFO or its appropriateness for a specific pathological gait disturbance. Although many studies have taken into account the adjustment of AFO mechanical characteristics on the kinematics and kinetics of the ankle, knee, and hip (8, 9, 11, 13, 15, 22, 31–42), a comprehensive method to adjust the mechanical characteristics of articulated AFOs to address specific observed pathological joint kinematic deviations has not yet been developed.

In clinical practice, there are two fundamental characteristics of an AFO that are commonly considered and adjusted to influence gait biomechanics. One is the AFO's resistance to ankle motion and the other is its ankle alignment angle. The AFO alignment angle is defined as the angle in the sagittal plane between the axes of the footplate and tibial sections without external force applied. A “neutrally aligned” AFO is defined as an AFO with a 90° ankle alignment angle. This alignment is also commonly referred to as a 0° alignment in vernacular terms, indicating that the sagittal plane tibial axis of the AFO is vertically aligned.

The resistance of an AFO is typically measured as the torque or bending moment in Newton-meters (Nm) that the AFO applies to resist ankle motion. The terms resistance and stiffness are sometimes used interchangeably; however, the stiffness of an AFO is more rigorously defined as the change of resistance per unit of ankle articulation and is typically measured in Newton-meters per degree (Nm/deg). Various devices have been developed to measure the stiffness of AFOs (43). These three AFO mechanical characteristics, namely, alignment, resistance, and stiffness, influence ankle motion in distinct ways, though the influence of stiffness as opposed to overall resistance is not yet fully understood. However, when adjusted these AFO characteristics can help mitigate pathologic gait abnormalities for patients with neuromuscular disorders (11, 12, 14).

Several recent studies have compared the biomechanical influence of AFOs with mechanical characteristics systematically adjusted to the unique needs of each individual patient (8, 11, 32–37, 41). Kobayashi et al. evaluated the influence of plantarflexion spring stiffness of articulated dorsiflexion assist-type AFOs and demonstrated a systematic effect on sagittal ankle position at initial contact and subsequent ankle motion throughout the gait cycle in individuals post-stroke (11).

Kobayashi et al. assessed sagittal ankle and knee motion and moments during walking using articulated dorsiflexion assist-type AFOs with varying levels of stiffness (37). Their work showed that for individuals post-stroke with knee hyperextension, this pathologic gait deviation could be ameliorated by increasing plantarflexion spring stiffness. This adjustment encouraged a heel-toe gait pattern at initial contact, resulting in a shifted ankle position toward dorsiflexion rather than plantarflexion, and a dorsiflexor moment at the ankle during early stance. Increasing plantarflexion stiffness also reduced the peak knee flexor moment and knee hyperextension by restricting shank reclination during single-limb stance (37). Their work also showed a systematic increase in both ankle dorsiflexion and knee flexion angles with increased plantarflexion-resist spring stiffness throughout the gait cycle. However, it is important to note that while group results suggested a systematic relationship between stiffness and effects, individual responses to varying stiffnesses were non-linear and specific to each subject.

It remains unclear whether it is most beneficial for an AFO to provide sufficient resistance to plantarflexion to maintain a fixed ankle position throughout the swing phase, while also limiting the maximum plantarflexion resistance to allow ankle plantarflexion at initial (heel) contact where the ground reaction force increases the external plantarflexion moment at the ankle.

Waterval et al. studied the influence of PLS AFOs with five different stiffnesses for 37 participants with neuromuscular disorders and non-spastic calf muscle weakness (15). PLS AFOs initially provide zero resistance to ankle motion. Their resistance to ankle motion is derived from the deflection of the footplate away from the ankle alignment angle, and so ankle alignment may change throughout swing phase. In this study on optimal walking economy, the stiffness of AFOs was highly individualized. A stiffness of 4.3 ± 0.5 Nm/deg was most frequently associated with the best gait economy, but this was observed only in 11 out of 37 participants. The most economical gait was achieved with AFOs having different stiffnesses: 2.8 ± 0.4 Nm/deg in eight participants, 3.5 ± 0.4 Nm/deg in six participants, 5.3 ± 0.7 Nm/deg in five participants, and 6.6 ± 1.1 Nm/deg in six participants. The least efficient AFO stiffness was observed most frequently at 6.6 ± 1.1 Nm/deg in 14 participants and at 5.3 ± 0.7 Nm/deg in 12 participants. Their results demonstrated that AFO stiffness individualized for each participant in the study reduced the energy cost of walking by 11% when compared to the stiffest AFO. It was hypothesized that the stiffest AFO would produce the greatest push-off energy based on calculation of energy stored and lost through bending moment hysteresis, but the stiffest AFO did not result in significantly lower mean walking energy cost (15). These results suggested that adjustment of PLS AFO stiffness for each individual patient is likely more beneficial than simply making an AFO stiffer. It should be noted that this study employed single stiffness settings for each of the different AFOs, with these settings remaining constant throughout the gait cycle. In practice, this approach is difficult to employ for individuals with complex neuromuscular pathologies where ankle motion is dysfunctional at certain points in the gait cycle but functional at other points. Even if a set of prefabricated AFOs with a range of stiffnesses were available at fitting, an appropriate stiffness AFO would still be difficult to prescribe because the guiding outcome of this approach is gait economy, which requires complex metabolic testing with portable O_2_ and CO_2_ sensor systems. Therefore, it is unlikely that this approach would be applicable to routine orthotic care in the clinical setting due to its expense, time, and effort, and the limitations of the orthotist's scope of practice and experience with respect to energy cost diagnostics. The method would also be susceptible to errors due to confounding variables such as food consumed prior to the test and the difficulty of achieving a steady state during walking.

## 2 Optimal mechanical characteristics of AFOs

### 2.1 Customary orthotic practice and challenges in AFO optimization

Determining the optimal mechanical characteristics of an AFO is a complicated task for both the prescribing physician, and the orthotist responsible for providing orthotic care and adjusting the AFO to improve patient ambulatory function. AFO designs that incorporate an adjustable ankle joint rather than requiring an irreversible change to the orthotic design to alter stiffness offer the ability to modify the AFO's mechanical characteristics quickly and reversibly in a clinical setting. These adjustable orthoses also facilitate the adaptation of those characteristics to the patient's evolving needs over time. Adjustability also offers the ability to change the AFO's mechanical characteristics progressively, and iteratively to achieve specific functional objectives. However, optimization requires that the goals of adjustment are clearly defined. In practice, it is often also necessary to prioritize and reconcile optimization goals considering a myriad of competing concerns in orthotic patient management.

The overall aim of AFO optimization is to reduce specific pathologic gait deviations. It is reasonable to assume that the reduction of pathologic gait deviations will improve patient ambulatory function (24–26, 28); therefore normal gait is often used as a comparative reference for adjustment. The adjustment process is typically informed by subjective and objective clinical indicators, e.g., patient verbal feedback and observation of the patient walking, respectively. It is widely accepted that three-dimensional (3D) instrumented gait analysis, including kinetic and kinematic data, is the gold standard of gait assessment. However, this type of motion analysis has limited availability and is costly, time-consuming, and complicated, which makes it impractical in many clinical settings. As a result, customary orthotic practice often relies on basic clinical techniques to evaluate objective clinical indicators. One such technique is the identification of gait deviations through observational gait analysis.

Several studies have demonstrated that observational gait analysis can result in substantial errors when used to identify gait deviations (44–47). However, studies also suggest that if the observer's attention can be focused on a few discrete gait events and the assessment is repeated multiple times, the ability of the observer to reliably identify gait deviations may be improved (48). The use of slow-motion video as an adjunct to observational gait analysis may also help improve the reliability of identifying gait deviations. Therefore, it is possible to improve the accuracy of identifying the orthotic influence on patient gait through iteration of AFO adjustments using repetitive observations of specific gait events with slow-motion video. This is typically done by pausing the motion and scrolling the video repeatedly through the gait event. Establishing the reliability of observational gait analysis is essential if it is to be used to determine whether a specific gait deviation has been reduced or increased through the adjustment of the AFO’s mechanical characteristics. Various gait assessment scales have been developed that utilize this concept (49). For example, the Edinburgh Visual Gait Score showed 69% agreement with 3D computerized gait analysis for maximum ankle dorsiflexion in stance, 83% agreement with maximum ankle dorsiflexion in swing, but only 47% agreement with peak knee extension in stance (49). While these observational gait tools may not achieve the same level of accuracy or precision as the gold standard of instrumented motion analysis, they can potentially improve the reliability, sensitivity, and validity of visual gait analysis when instrumented analysis is not feasible.

Therefore, by focusing on a few key gait characteristics, the orthotist's ability to identify a patient's gait deviations reliably and validly may be improved, and by doing so, observational gait analysis may be adequate for the purpose of AFO adjustment and optimization of patient ambulatory function. However, it should be noted that substantial errors may be associated with the less rigorous application of observational gait analysis to AFO optimization. Therefore, an iterative approach to the change of an AFO’s mechanical characteristics with repetitive observation is essential to minimize observational errors if the assessment is to be applied to orthotic practice.

### 2.2 AFO mechanical characteristics

To reduce pathologic gait deviations, the intrinsic sagittal plane mechanical characteristics of an AFO should be adjusted. As aforementioned, these intrinsic mechanical characteristics are the AFO's alignment, resistance, and stiffness. Non-articulated AFOs typically exhibit high structural stiffness, ranging between 8 and 18 Nm/deg, depending on the fabrication method, design, and materials used (50). Following fabrication, the structural stiffness of a non-articulated AFO is fixed unless its shape is permanently changed. The resistance of high stiffness AFOs increases rapidly with deflection of the AFO footplate, although their initial resistance is 0 Nm. By contrast, traditional articulated AFOs use mechanical ankle joints to resist ankle motion. These orthotic components typically resist ankle motion using internal springs with stiffness that is significantly lower than the structural stiffness of a solid AFO. The stiffness of these traditional hinged AFOs may be on the order of 0.25 Nm/deg (11, 35, 39). Traditional hinged AFOs initially present 0 Nm of resistance to ankle motion, and because of their relatively low stiffness, they may only be suitable for managing swing-phase gait abnormalities (e.g., foot drop), where the resistance required to influence pathologic gait is relatively low compared to stance phase.

**Figure 2:**
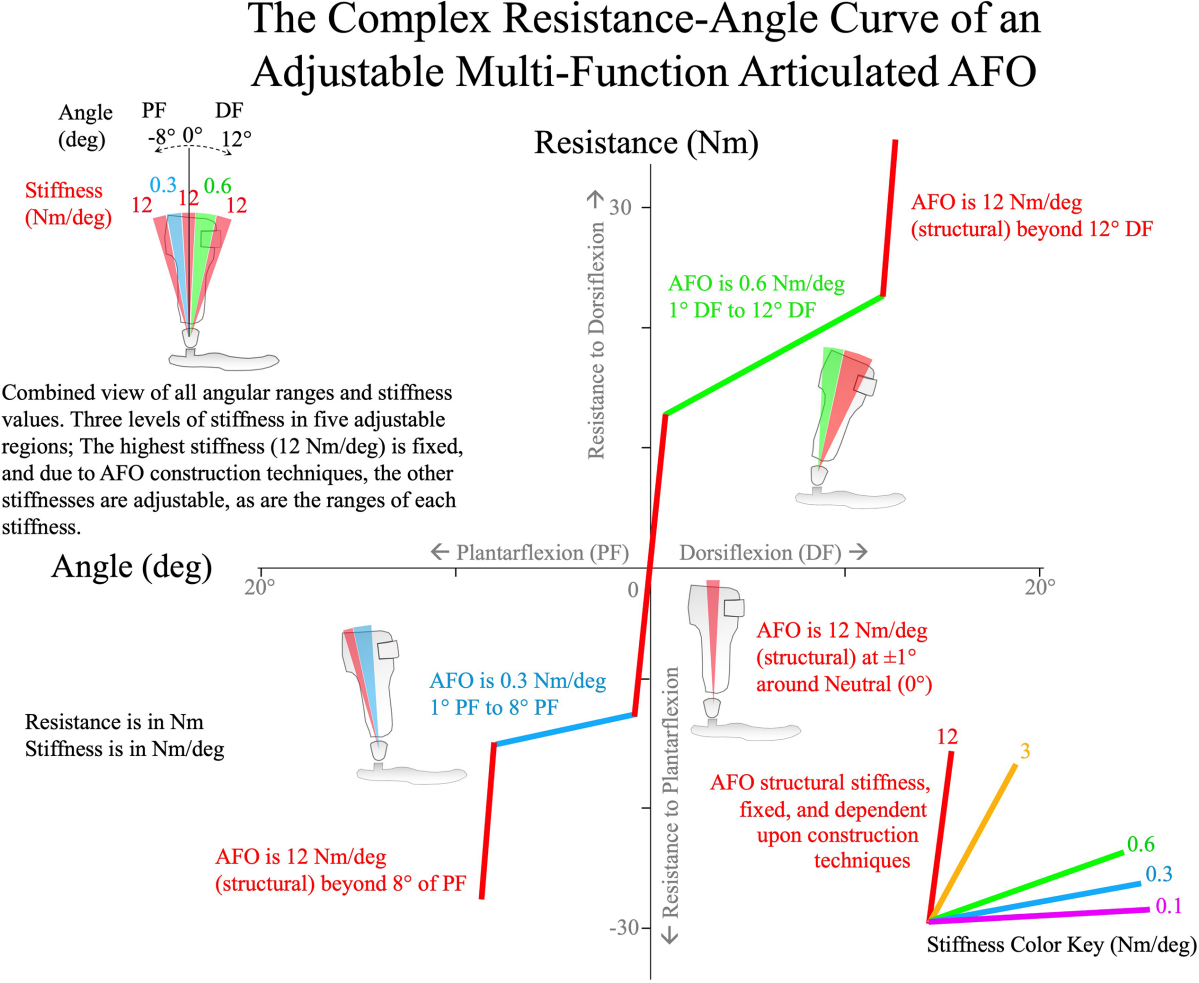
A resistance (torque, Nm) vs. angle (deg) plot of an example multi-function articulated AFO with five adjustable ranges with different stiffnesses. Two of these stiffnesses are adjustable, and the high stiffness is due to fabrication methods. The AFO cartoon in the upper left shows the total range of the AFO with specific ranges colored to represent different stiffnesses.

**Figure 3:**
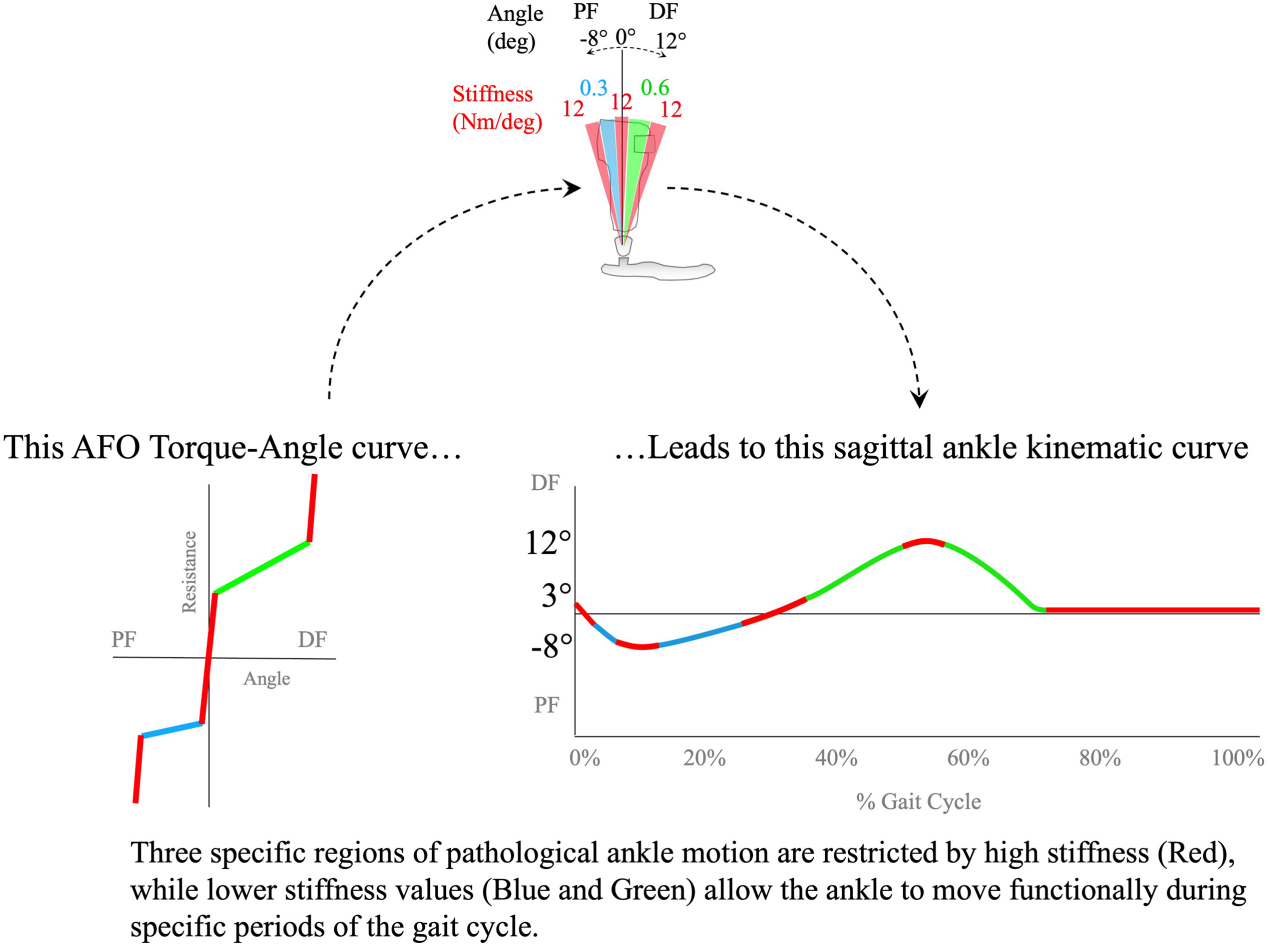
A typical multi-function articulated AFO's Resistance vs. Angle curve results in a specific sagittal ankle kinematic curve. The limits to motion caused by the highest stiffness are represented in red (12 Nm/deg) mainly during swing phase in the curve, with blue representing a low stiffness of 0.3 Nm/deg in early stance, and green representing a low stiffness of 0.6 Nm/deg in mid- and terminal stance. The red stiff region as the ankle crosses neutral provides stability in the single-limb stance, but allows ankle motion in both dorsiflexion and plantarflexion as the ankle angle changes.

An emerging class of articulated ankle-foot orthosis with features that facilitate improved control over AFO mechanical characteristics has been recently introduced to the orthotics profession. The first of these devices was the Neuro Swing double-acting ankle joint introduced by Fior and Genz (Lüneburg, Germany) in 2013. In 2016, Becker Orthopedic (Troy, Michigan, United States) introduced the Triple Action multi-function ankle component and in 2019 Otto Bock Healthcare (Duderstadt, Germany) introduced Nexgear Tango. These advanced orthotic components differ slightly in their feature set, but they all share the defining characteristics of multi-function orthotic ankle components. Multi-function articulated AFOs are well suited for managing both swing-phase and stance-phase gait deficits due to their high resistance to ankle motion and adjustability. The resistance and stiffness of multi-function articulated AFO springs are typically much higher than those found in traditional articulated AFOs. In addition, multi-function articulated AFOs offer the advantage of more precise adjustment, with mechanical characteristics that are de-coupled from one another. This allows for independent adjustment of mechanical characteristics in a way that is more versatile than traditional articulated AFOs. The stiffness of component springs can be changed to accommodate a broader range of patient weights, and these devices possess the unique feature of presenting a resistance threshold, or pre-load torque (Nm) to ankle motion. The resistance threshold of a multi-function articulated AFO is the torque necessary to move the AFO footplate away from its alignment angle against the ankle joint springs. When the torque applied to the AFO footplate is below the resistance threshold, the multi-function articulated AFO presents the high structural stiffness of the orthosis to resist ankle motion and the footplate deflects minimally as would a much higher stiffness non-articulated AFO. However, when the external ankle moment exceeds the resistance threshold of the ankle component, the footplate begins to move away from its ankle alignment angle and the resistance of the AFO continues to increase at a rate determined by the stiffness of the ankle joint springs. This stiffness is typically less than the structural stiffness of a non-articulated, e.g., solid AFO, but significantly higher than the stiffness of a traditional articulated AFO. The maximum range of ankle motion is also adjustable, and when this motion limit is reached, the AFO again presents high structural stiffness to resist ankle motion (Figure 2). Therefore, the total resistance that a multi-function articulated AFO applies to influence ankle motion is determined by its structural stiffness, resistance threshold, and dorsiflexion and plantarflexion spring stiffnesses. This mechanical behavior results in a complex resistance vs. angle curve resembling a sigmoid that exhibits varying stiffness throughout specific and adjustable ankle ranges of motion (Figure 3).

This complex behavior of a multi-function articulated AFO allows functional ankle motion against reduced stiffness while resisting motion through dysfunctional ranges. The multi-function articulated AFO also facilitates the independent adjustment of ankle alignment angle without altering the resistance or stiffness settings (36, 40).

## 3 Development of an evidence-guided algorithm

### 3.1 Evidence-guided algorithm for the adjustment of multi-function articulated AFOs in the clinical setting

There is a lack of standardized orthotic adjustment algorithms in orthotic practice. One orthotic algorithm described by Owen involves the optimization of AFOs combined with shoe outsole modification to improve patient ambulatory function. The AFO footwear combination (AFO-FC) is clinically “tuned” by modifying the shoe outsole shape to improve gait in children with cerebral palsy (CP) (51). This method of adjusting the AFO-FC has also been described by Jagadamma et al. for use in post-stroke adults with hemiplegia (22). The method initially focuses on determining the ankle alignment angle by evaluating the patient's passive range of ankle dorsiflexion before fabrication of the rigid AFO. With the rigid AFO and shoes fitted to the patient, optimization for standing balance and knee position is accomplished by adjustment of the heel height of the shoe. The shape of the heel and forefoot rockers of the shoe outsole are subsequently adjusted by abrasive grinding to “tune” the shape of the shoe outsole, reducing pathologic shank and thigh kinematics in both early and late stance phases of gait. The stiffness of an AFO footplate may affect gait patterns as well (52). Owen's method thus focuses on the reduction of pathologic shank and thigh gait deviations with an emphasis on observing these limb segments with respect to the vertical axis.

In contrast to Owen's work, the adjustment algorithm proposed in this present work aims to preserve functional ankle motion while reducing pathologic ankle and knee gait deviations. This algorithm is novel as it is focused on adjusting the mechanical characteristics of a multi-function articulated AFO to associate with and systematically influence specific events throughout the gait cycle (8, 11, 36–42, 53, 54).

**Figure 4:**
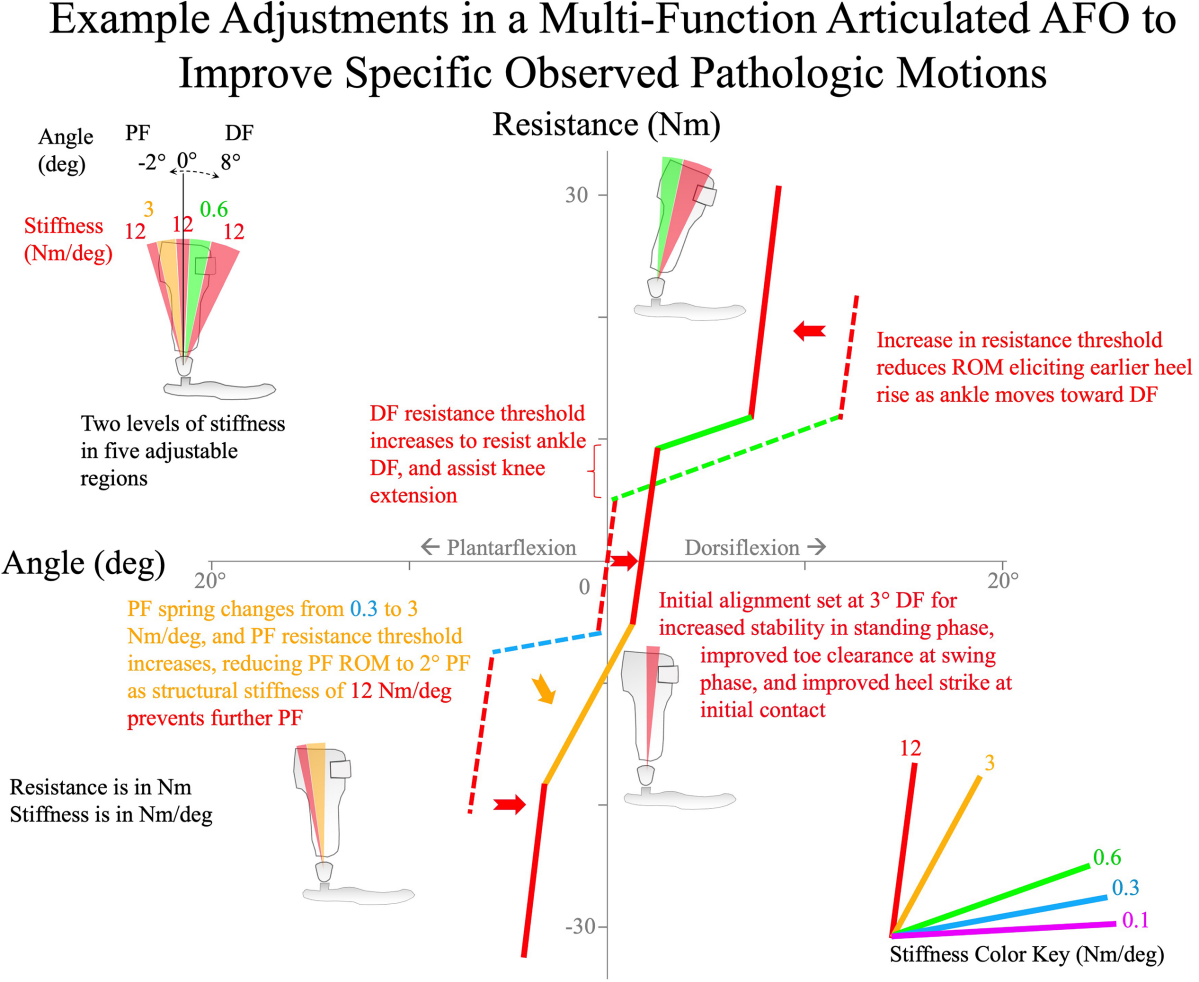
A demonstrated series of adjustments to stiffness and resistance threshold of a multi-function articulated AFO to treat specific observed pathologic ankle motions. The overall outcome is a stiffer AFO with narrower bands of low stiffness ranges.

Multi-function articulated AFO mechanical characteristics have been found to systematically influence gait kinematics and kinetics of the ankle and knee (8, 11, 33–39, 41). Studies demonstrate that changes to the AFO ankle alignment angle influence ankle angle throughout the gait cycle (8, 11, 33, 34). Studies also demonstrate that resistance to ankle plantarflexion systematically influences ankle and knee sagittal kinematics and kinetics throughout the swing phase and during the first rocker of gait (31, 39). Resistance to ankle dorsiflexion systematically influences the second rocker of gait, mid-stance to pre-swing (36). Evidence also suggests that this influence is mostly isolated, facilitating the association of specific AFO adjustments with particular phases of the gait cycle. Therefore, the algorithm was developed to exploit this isolated influence of AFO adjustments to help establish a clear pathway toward optimization, providing guidelines to associate observed gait deviations with specific multi-function articulated AFO adjustments while remediating undesirable, iatrogenic consequences of the orthotic treatment. Examples of the adjustments that can be made to a multi-function articulated AFO are shown in Figure 4, where each resistance threshold value is adjusted in response to an observed gait deviation.

The algorithm was developed to be used in the clinical setting, where access to a sophisticated gait lab is typically not available. The method relies on observational gait analysis augmented by repeated observation of specific gait events using slow-motion video to increase the reliability of observations and indicated adjustments. Contemporary smartphones equipped with high-resolution slow-motion cameras make this feasible in a clinical setting. Observational gait analysis may be further improved by capturing video from different perspectives, e.g., both the sagittal and coronal planes, which may also be helpful to detect changes in gait characteristics as well as to estimate joint angles or step lengths.

A specific and clinically relevant set of gait events was selected for the adjustment algorithm (Supplementary File 1) based on reliability of identification as well as clinical utility:

1. Knee position and shank inclination in static weight bearing.
2. Perceived weight line with respect to ankle, knee, and hip joint anatomical axes in static weight bearing.
3. Toe clearance in mid-swing.
4. Knee extension at terminal swing.
5. Foot position at initial contact.
6. Knee kinematics through the first rocker.
7. Tibial progression through the second rocker.
8. Heel rise at terminal stance through the third rocker.
9. Knee kinematics after mid-stance.
10. Step length and step length symmetry.

Pathologic deviations of these specific gait events inform associated adjustments to multi-function articulated AFO mechanical characteristics. Evidence to support the systematic effects of AFO adjustment intended to influence specific gait characteristics is supported by cited literature in the text and Figures 5–13.

**Figure 5:**
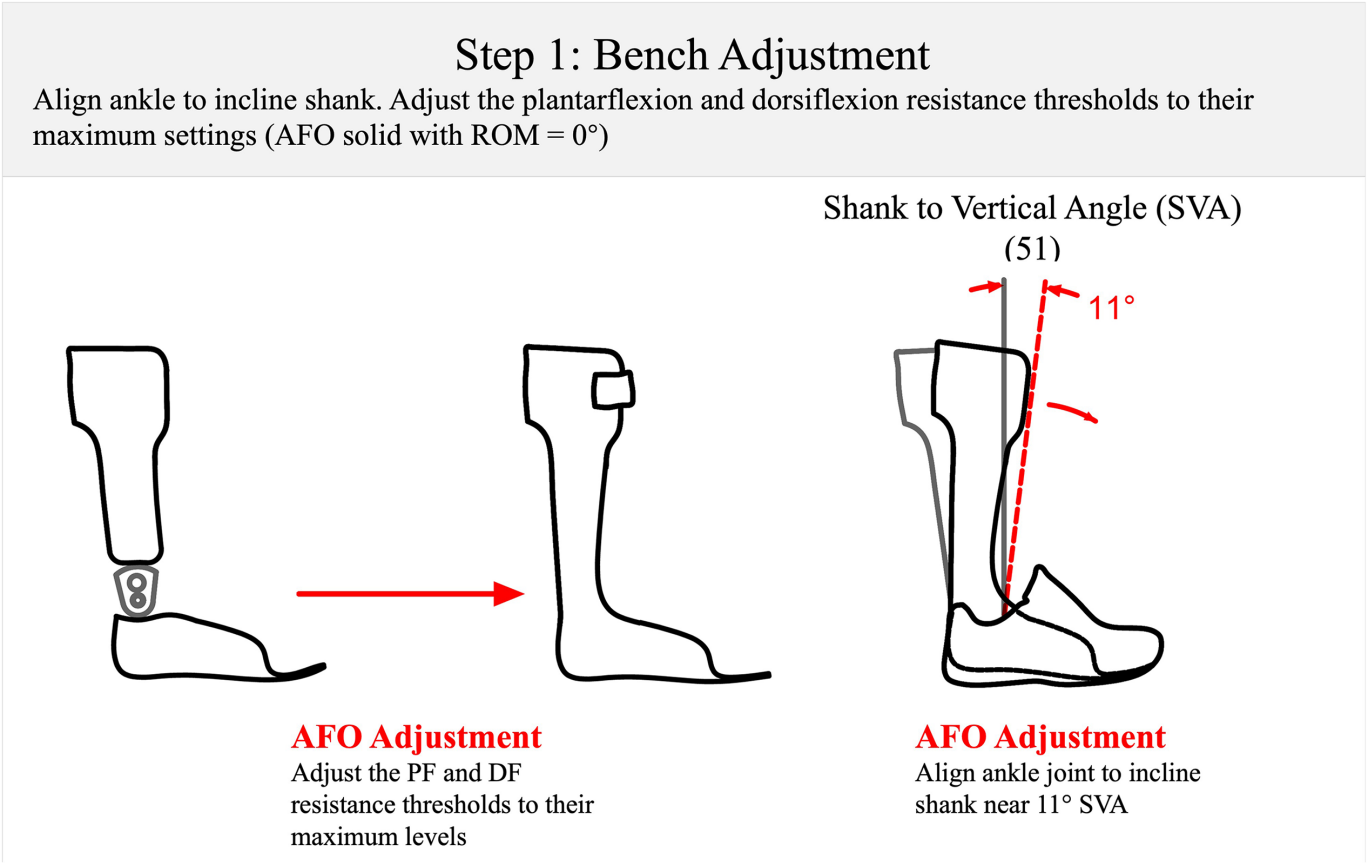
Step 1: the bench adjustment of a multi-function articulated AFO at the start of the optimization process. The AFO is set at an incline of 11° of SVA (51) to accommodate a typical shoe. The PF resistance threshold and DF resistance threshold are adjusted to maximum. SVA, shank to vertical angle; PF, plantarflexion; DF, dorsiflexion.

**Figure 6:**
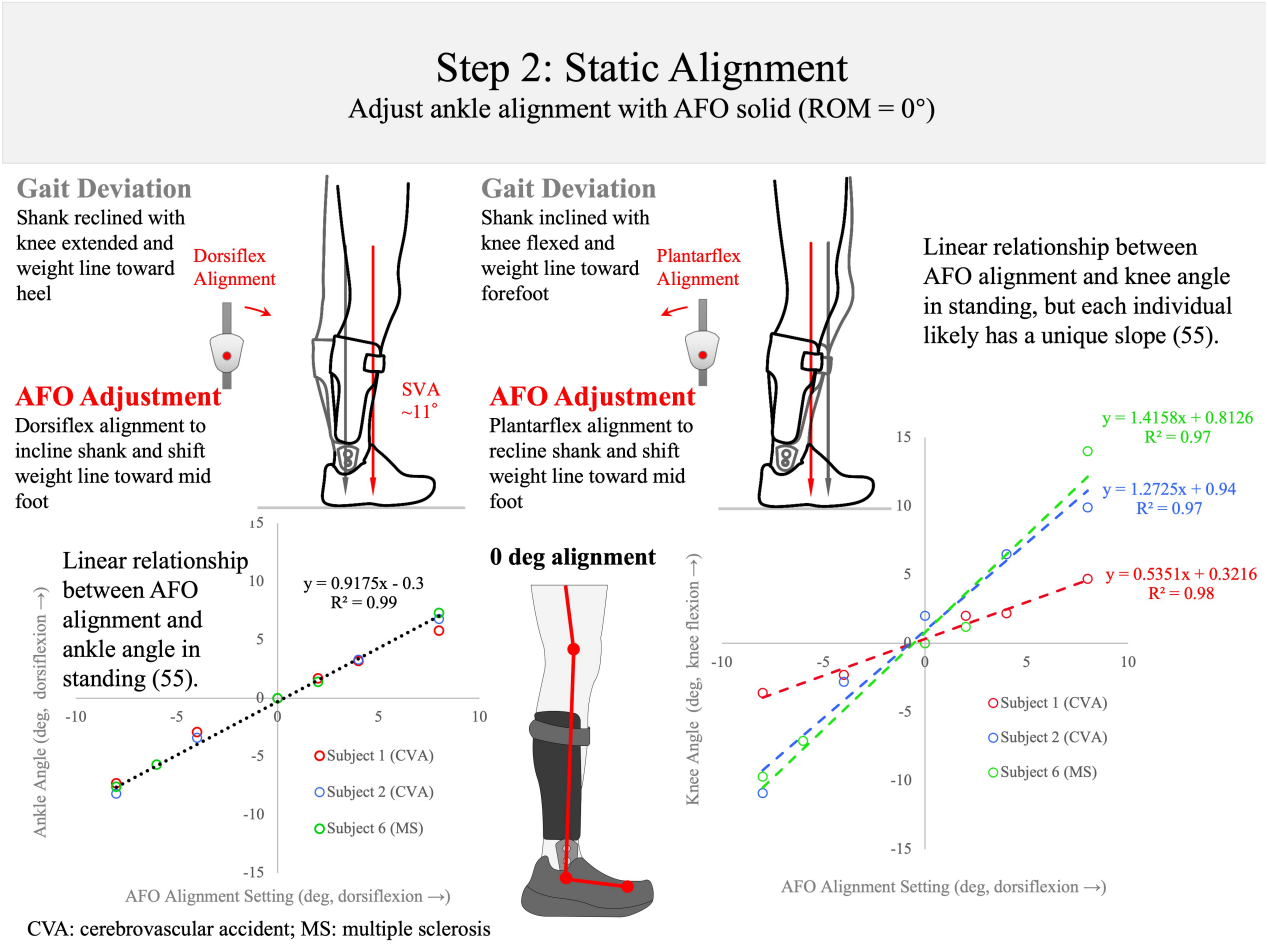
Step 2: static alignment. With the AFO range set to 0° of plantarflexion and 0° of dorsiflexion (range of motion: ROM = 0°), the alignment angle of the AFO is set so that the center of mass weight line falls at the middle of the foot (red line). The gray line shows when the AFO is too plantarflexed (left) or dorsiflexed (right). Previous research has shown that the standing ankle angle responds systematically to AFO alignment angle changes, accounting for >99% of the participant's variance (55). The knee also shows a linear relationship to AFO alignment angle (*R*^2^ > 0.96), but each individual likely has a unique slope of knee position in standing as AFO alignment angle is altered (55).

**Figure 7:**
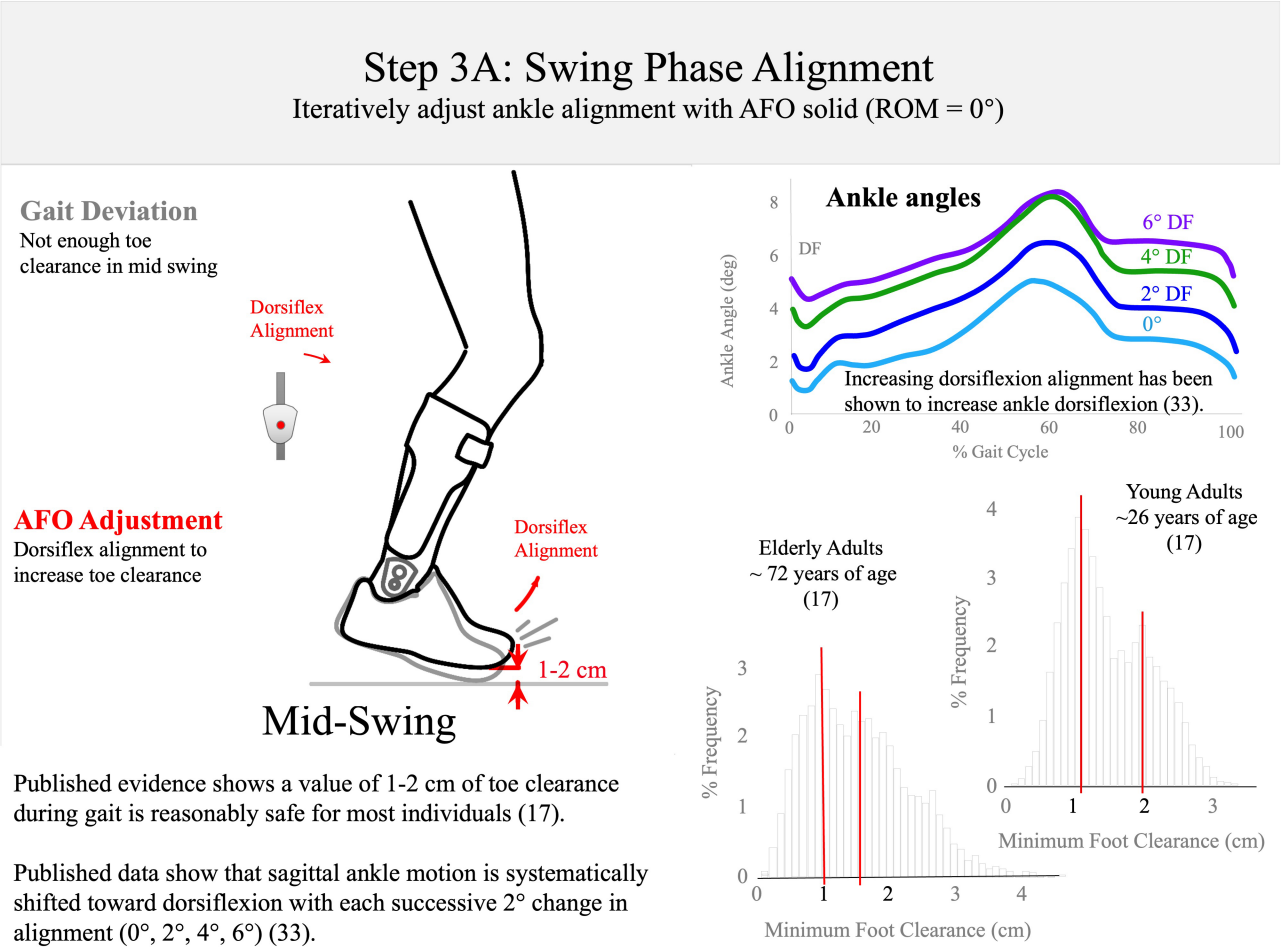
Step 3A: swing-phase alignment. The AFO alignment should be dorsiflexed to create 1–2 cm of toe clearance in mid-stance (17). Published data show that sagittal ankle motion is systematically shifted into dorsiflexion with each successive 2° alignment change (0°, 2°, 4°, 6°), adapted from reference (33).

**Figure 8:**
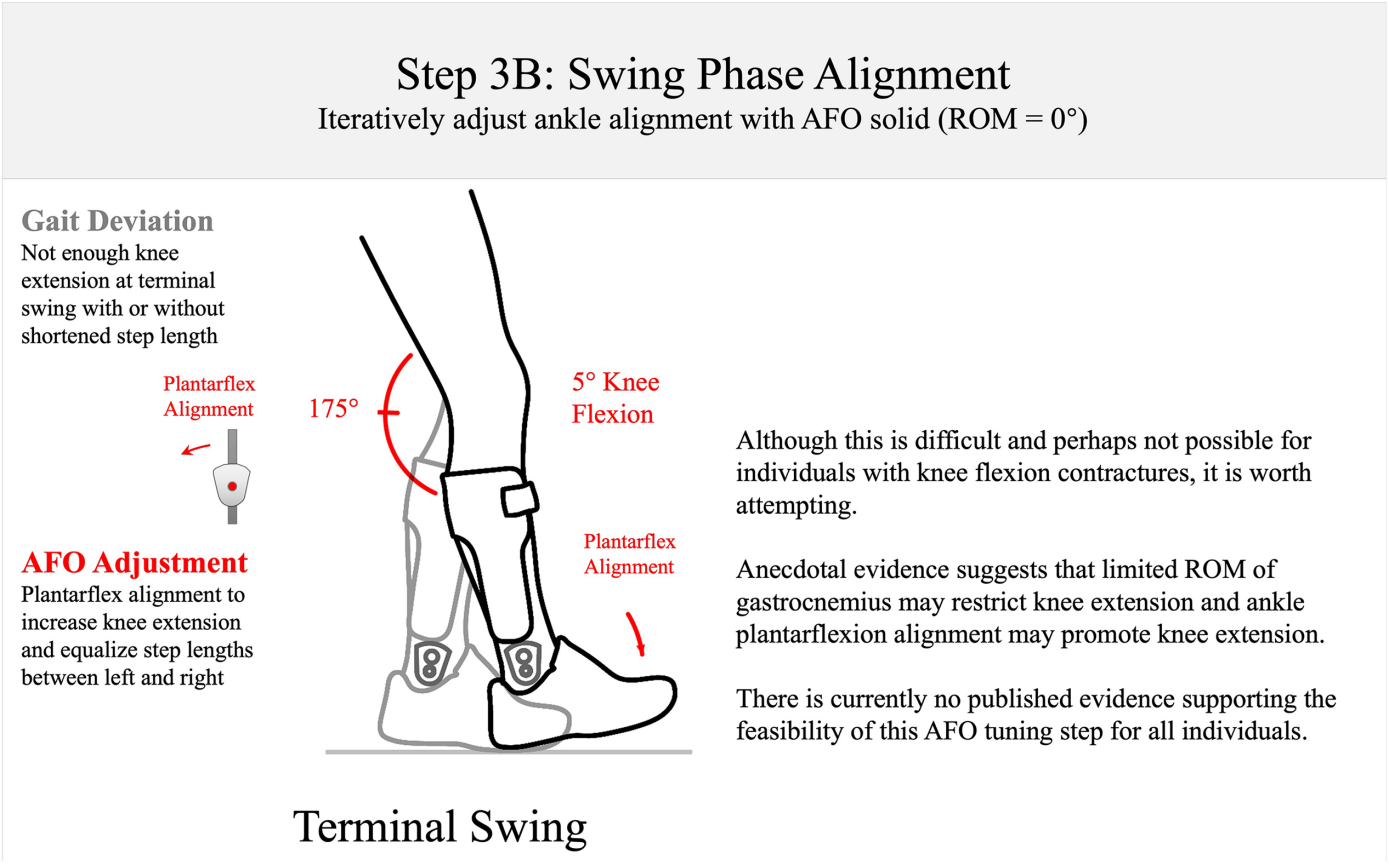
Step 3B: swing-phase alignment. The AFO should be aligned to encourage near full knee extension at initial contact. This is a challenging goal, and there is as yet no published evidence to support this goal in optimization of AFOs.

**Figure 9:**
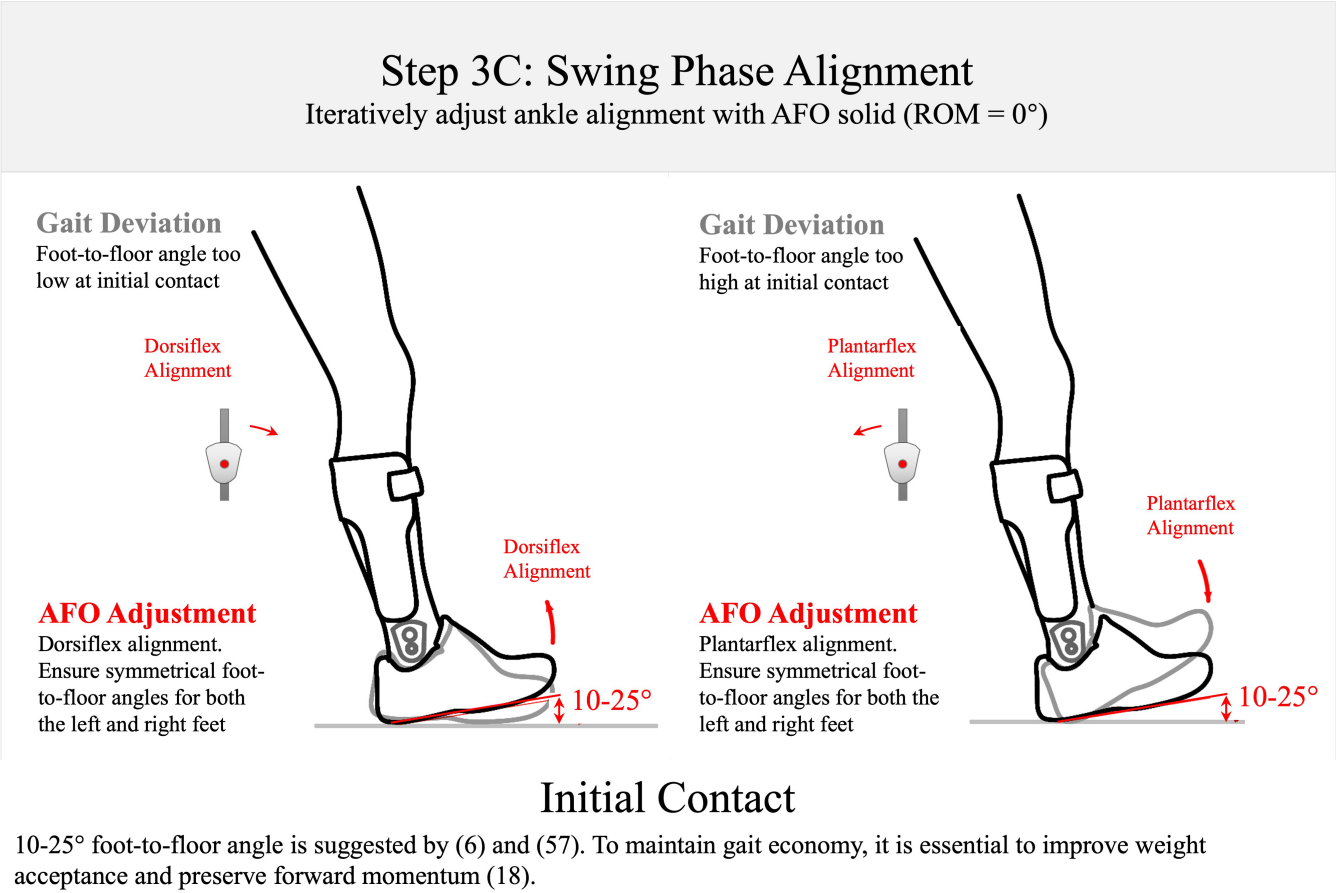
Step 3C: swing-phase alignment. The AFO alignment should be adjusted to create a 10°–25° between the foot and the floor at initial contact. 10°–25° foot-to-floor angle is supported by the studies by Perry and Vette et al. (6, 57). Lower angles than this are observed in pathological individuals leading to foot-flat or toe-heel contact (57) that can disrupt weight acceptance and forward momentum preservation in early stance (18).

**Figure 10:**
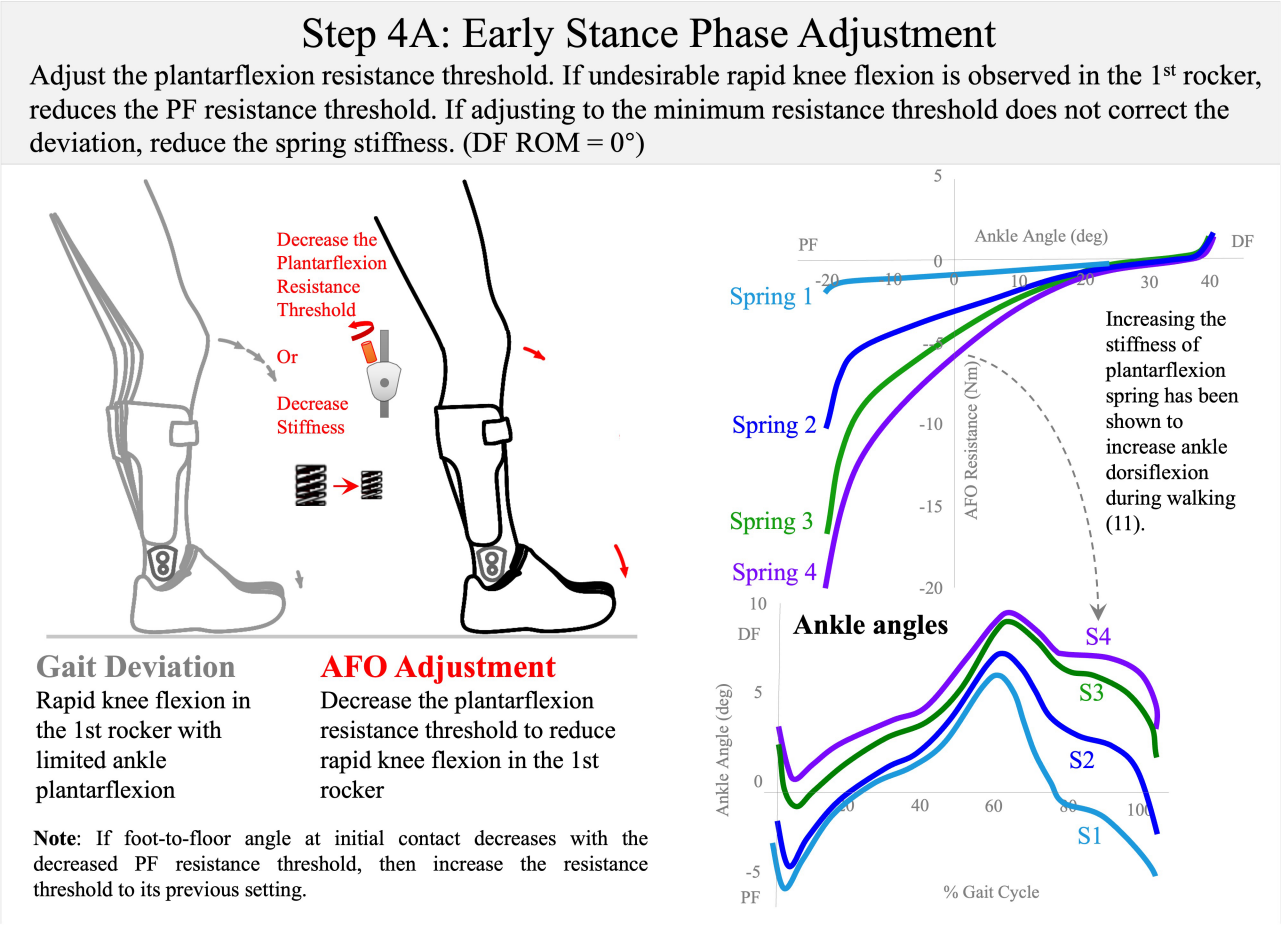
Step 4A: early stance-phase adjustment. The AFO plantarflexion range should be adjusted or the spring stiffness changed to allow the first rocker with controlled knee flexion. Dorsiflexion range of motion is set to 0° (DF ROM = 0°). Published data have demonstrated that sagittal ankle motion can be systematically shifted into plantarflexion or dorsiflexion by altering plantarflexion range spring stiffness (11). To control rapid knee flexion in early stance, either decrease the plantarflexion resistance threshold or choose a less stiff spring (3 Nm/deg → 0.6 Nm/deg → 0.3 Nm/deg).

**Figure 11:**
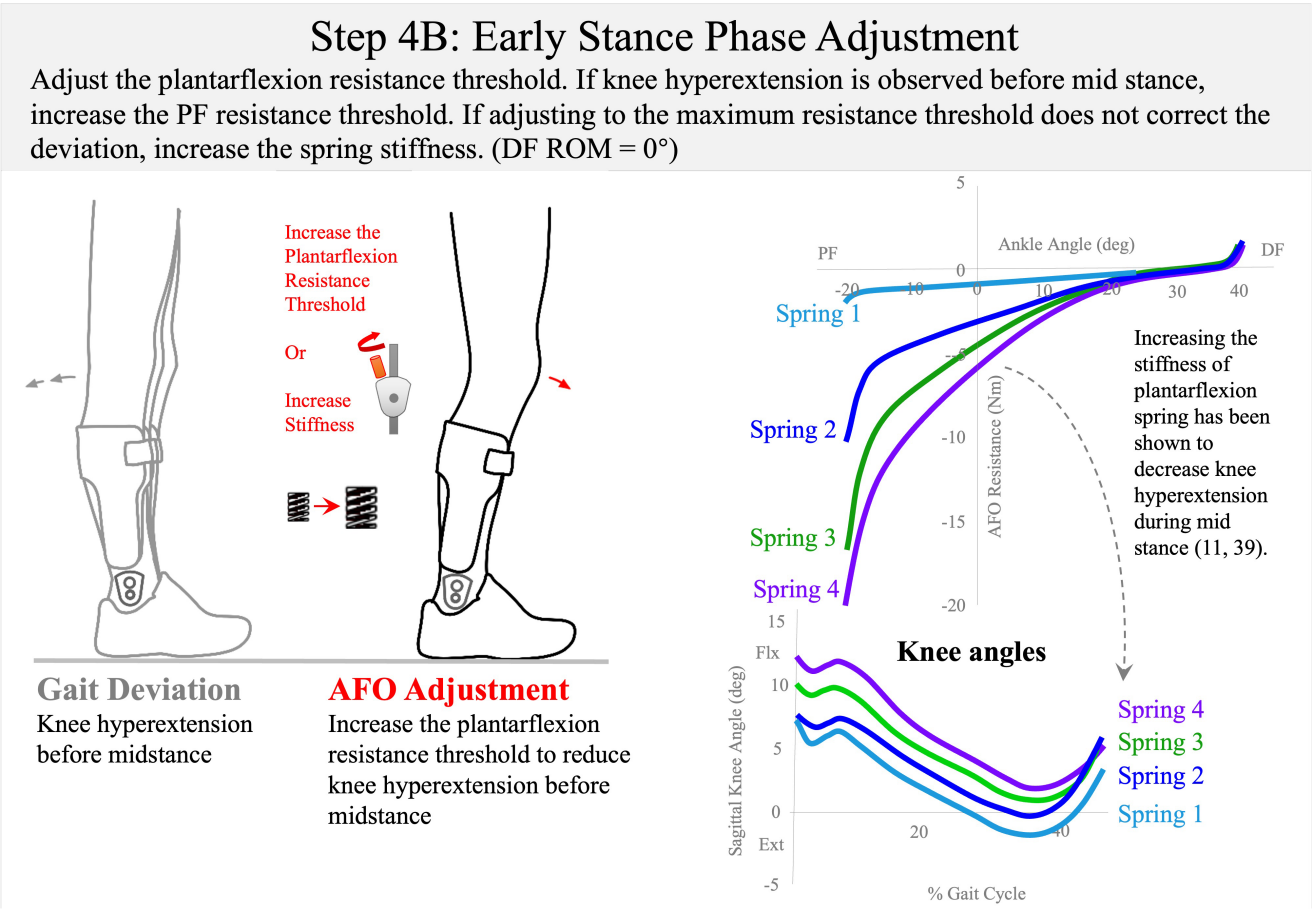
Step 4B: early stance-phase adjustment. Published data have shown that a more compliant spring can shift the knee to more extension in early stance (11, 39). To control knee hyperextension in early stance, either increase the plantarflexion resistance threshold or choose a stiffer spring (0.3 Nm/deg → 0.6 Nm/deg → 3 Nm/deg).

**Figure 12:**
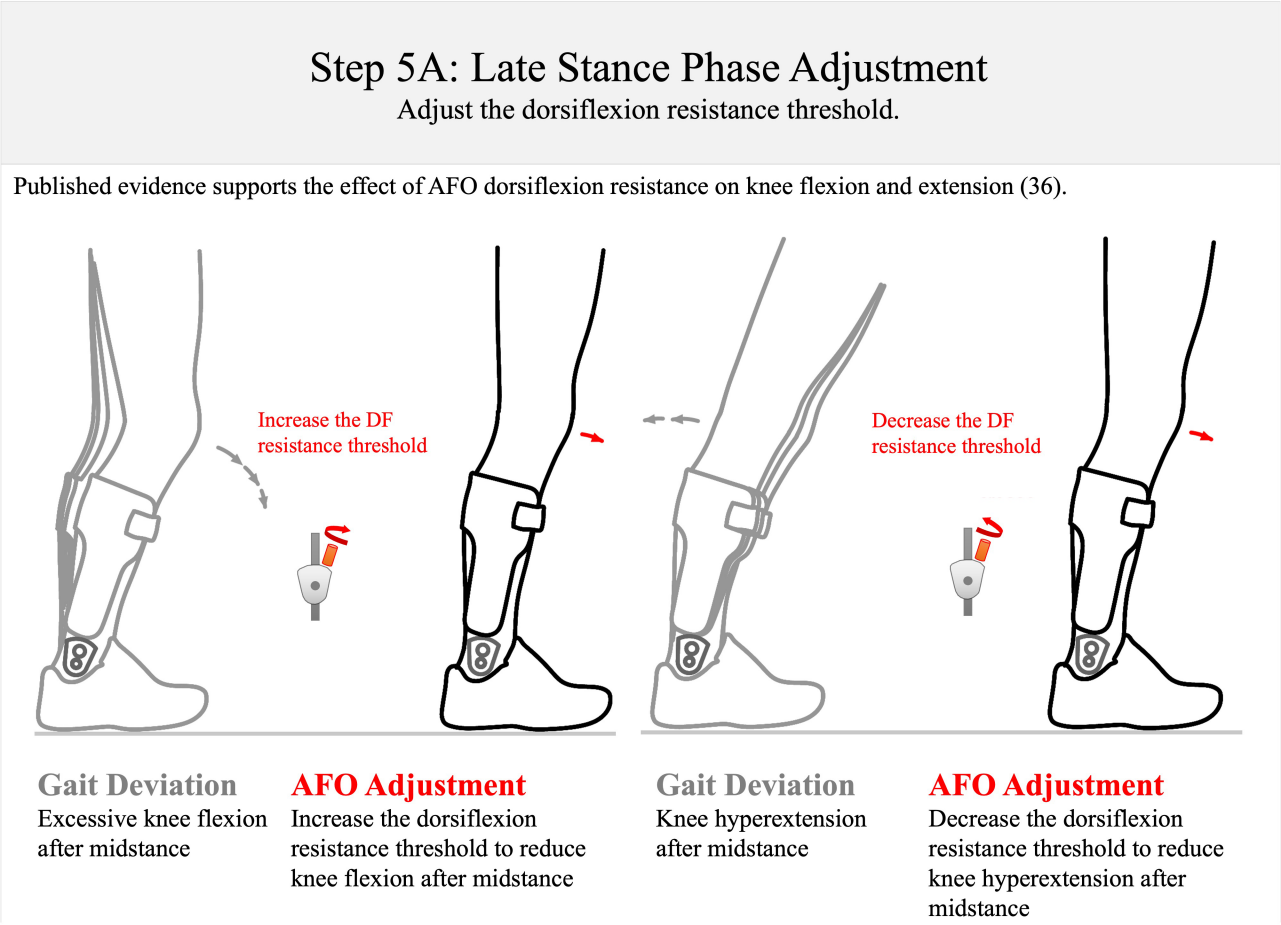
Step 5A: late stance-phase adjustment. To reduce excessive knee flexion in stance, the DF resistance threshold should be increased; to control knee hyperextension in the single-limb stance, the dorsiflexion resistance threshold should be decreased. This approach is supported by data from Ref. 36. DF, dorsiflexion.

**Figure 13:**
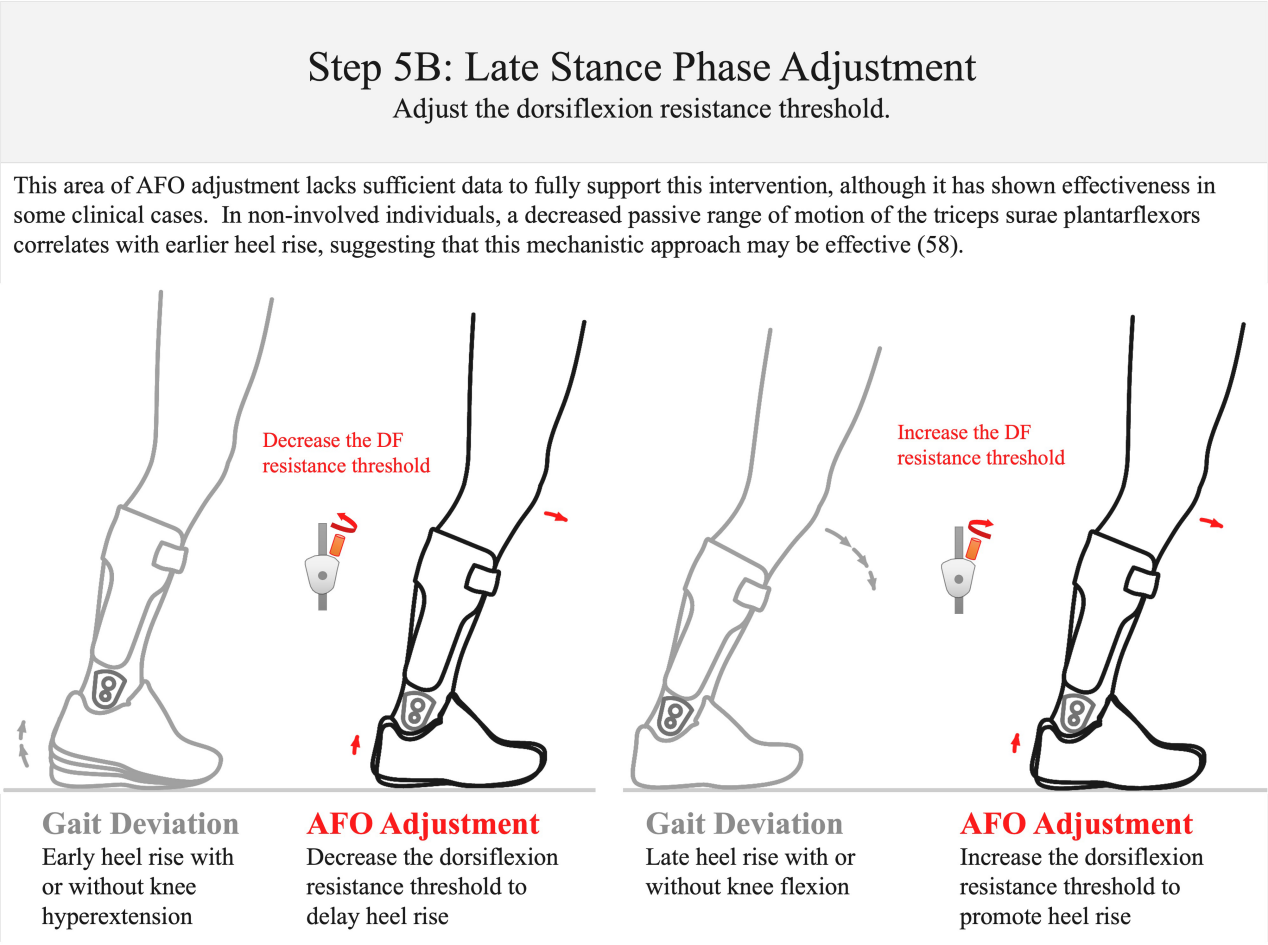
Step 5B: late stance-phase adjustment. If heel rise occurs too early in the gait cycle, decrease the DF resistance threshold. If the individual exhibits late heel rise with hyperdorsiflexion, increase the dorsiflexion resistance threshold (58). There is as yet no published evidence that supports or refutes this adjustment. DF, dorsiflexion.

Throughout the subsequently described process, the term *alignment* signifies changing the AFO ankle alignment angle without adjustment of AFO resistance threshold or stiffness, while the term *adjustment* is used to indicate a change of resistance threshold or component stiffness with or without a change of alignment. As previously described, the resistance threshold of the multi-function articulated AFO is adjusted by pre-compressing, or pre-loading the component springs within the ankle joint. AFO component stiffness is adjusted by installing different springs or combinations of springs in the ankle joint, and may scale the range of resistance threshold adjustment according to the weight and biomechanical deficits of the patient. Steps 4 and 5 in the following procedure involve initially setting the AFO resistance range by adjusting spring stiffness, followed by adjusting the resistance threshold to reduce the observed pathologic gait deviations.

### 3.2 The multi-function articulated AFO adjustment algorithm—the total time to execute the algorithm is approximately 30 min

*Step1: bench adjustment (orthosis on the bench)—approximately 3 min*

Bench adjustment involves setting the mechanical characteristics of the orthosis to an initial condition in preparation for optimization. The term and procedure are similar in some aspects to the more familiar “bench alignment*”* originally coined by prosthetists. Prosthetic bench alignment of a transtibial prosthesis refers to the process of adjusting the initial alignment of the prosthetic socket with respect to the prosthetic foot. While there is an accepted standard for prosthetic bench alignment, accommodation is typically made to the socket angle in cases where the patient has a flexion contracture or atypical joint alignment of the residual limb, and for the anticipated heel height of the shoe.

By contrast, orthotic “bench adjustment” in the algorithm implies setting the initial AFO ankle alignment angle to slightly incline the patient's shank with the AFO and shoe donned (Step 1: Figure 5) and adjusting the resistance of the AFO to “lock” the ankle joint, simulating the mechanical characteristics of a high stiffness, non-articulated AFO. This is done to achieve maximum stability and safety for the patient during “Static Alignment”.

*Step 2: static alignment (orthosis donned while patient is standing)—approximately 4 min*

Static alignment is performed with the AFO and shoes fitted to the patient in quiet standing (Step 2: Figure 6) and the AFO “locked” to simulate a non-articulated AFO. Static alignment changes the ankle angle with concomitant change to knee flexion. The goal of this step of the algorithm is to adjust the initial ankle alignment angle to achieve slight shank inclination and improve the patient's subjective sense of balance in quiet standing. If accommodation is necessary for a plantarflexion contracture to position the ankle within its passive range of motion (ROM), a heel lift under the AFO may be beneficial. During Static Alignment, an objective measure of 10°–12°  of shank inclination, e.g., 11°  shank to vertical angle (SVA), may be used as a starting point. This angle was originally determined by Owen to be the average shank to vertical angle for optimizing gait kinematics and kinetics in their method (51). Consideration should also be given to the position of the patient's weight line with respect to the imaginary line joining the trochanter, knee, and ankle (TKA). The patient's subjective feedback is critical during static alignment, and their sense of balance, stability, and comfort are assessed as part of this process. Again, a parallel can be drawn to the static alignment of a transtibial prosthesis, which includes anteriorly tilting the prosthetic socket (i.e., flexing the socket) and aligning the knee center anterior to the ankle axis such that the patient's weight line passes through the middle third of the foot (56). A previous study has shown that ankle and knee joint angles respond systematically to AFO alignment angle changes while standing, but each individual likely has a unique profile of knee position as the AFO alignment is changed (55).

*Step 3: swing-phase alignment (orthosis donned while patient is walking)—approximately 7 min*

When satisfied with the static alignment, the patient is asked to walk to adjust swing phase alignment (Step 3: Figures 7–9). This step of the algorithm is also performed with the ankle joint adjusted to simulate a non-articulated AFO. Published data show that the sagittal ankle angle is systematically changed with ankle alignment of the multi-function articulated AFO. The goal of the swing-phase alignment is to optimize ankle alignment to improve toe clearance in mid-swing, knee extension at terminal swing, and foot position and foot-position symmetry at initial contact. These three gait events are observed and prioritized during swing-phase alignment according to the following guidelines. During mid-swing, toe clearance is evaluated with a goal of achieving at least 1 cm of clearance between the shoe and the floor (Step 3A: Figure 7). A minimum toe clearance of 1–2 cm has been suggested for the young and elderly adults (16, 17). Ankle alignment may be adjusted toward dorsiflexion to increase toe clearance. Assuming the structural stiffness of the orthosis is sufficient, the kinematic response to this adjustment has been found to be systematic (33).

After alignment for toe clearance, knee extension at terminal swing is observed and compared with the normative value of 175° of the knee popliteal angle, or 5° of knee flexion at terminal swing (Step 3B: Figure 8). If the knee does not fully extend at terminal swing, it could be due to a knee flexion contracture or shortened gastrocnemius, which may be exacerbated by excessive ankle dorsiflexion alignment. If the knee does not achieve full extension, the previous objectives may need to be reconciled by further iterative adjustment of ankle alignment to achieve overall optimization. However, it should be noted that this last objective of full knee extension is not well supported by the published literature. Anecdotal clinical observations do suggest that it may have utility for orthotic optimization; therefore, it is included in the algorithm with the caveat that the measure should be cautiously utilized. However, the clinician should not rely solely on this observation for definitive decision-making during AFO optimization.

The angle between the shoe outsole and the floor at initial contact i.e., foot-to-floor angle, has been described by Perry in normal gait to be 25° at the time of heel strike (6). Vette et al. show a range of 15°–20° of foot-to-floor angle at initial contact (57). Therefore, a range of 10°–25° is used as the goal for swing-phase alignment of the foot position at initial contact and foot-position symmetry (Step 3C: Figure 9). Ankle alignment is optimized to achieve this goal by adjusting ankle alignment toward dorsiflexion or plantarflexion to increase or decrease the foot-to-floor angle, respectively.

To summarize, static- and swing-phase alignment is performed with the multi-function articulated AFO adjusted to its maximum resistance settings (against the dorsiflexion and plantarflexion motion limiting stops); therefore, any pathologic gait deviations observed during the adjustment of swing-phase alignment are reduced by optimizing ankle alignment to balance and prioritize concerns among the observed gait deviations. Toe clearance in mid-swing and foot position at initial contact are prioritized. However, if there is observed restriction of knee extension in terminal swing due to increasing dorsiflexion alignment of the AFO, particularly associated with a shortened gastrocnemius muscle, then optimizing toe clearance and/or foot position at initial contact becomes crucial. This optimization should consider how the knee flexion angle throughout the swing affects the position of the foot with respect to the floor.

Iteration between AFO settings for “Static Alignment” and “Swing-Phase Alignment” may be necessary to reconcile competing concerns between these two steps of the adjustment algorithm and to achieve the optimal alignment setting for balance in quiet standing with improved swing-phase gait mechanics. There may be a point of diminishing benefits to this compromise in reduction between gait deviations as the ankle alignment angle is changed. The algorithm relies on clinical judgment and iterative adjustments to alignment and careful, repeated observations to identify the optimal balance between these potentially competing concerns.

*Step 4: early stance–phase adjustment (orthosis donned while patient is walking)—approximately 8 min*

During static- and swing-phase alignments, the plantarflexion resistance threshold had been previously adjusted (during bench adjustment) to “lock” the ankle simulating a non-articulated AFO. In this configuration there was no concern that the orthosis would present inadequate resistance to prevent ankle plantarflexion through the swing phase because the orthosis presents the high structural stiffness of a non-articulated AFO to the ankle. However, with the patient walking in a maximally supportive AFO with high resistance to plantarflexion, undesirable rapid knee flexion in the first rocker may be observed (11, 34, 37, 39). This iatrogenic gait deviation is mitigated by reducing the plantarflexion resistance threshold in the next step of the algorithm (Step 4A: Figure 10).

Early Stance–Phase Adjustment involves reducing the plantarflexion resistance threshold to allow ankle plantarflexion in the first rocker when the ground reaction force from initial contact to loading response exceeds that resistance. When making this adjustment, it is important to maintain the plantarflexion resistance threshold high enough to maintain the ankle position at the ankle alignment angle throughout the swing phase until initial contact. The goal of adjusting the AFO resistance threshold for early stance phase is to encourage controlled knee flexion by permitting resisted ankle plantarflexion during the first rocker of the gait cycle. Therefore, the plantarflexion resistance threshold setting should permit ankle plantarflexion from initial (heel) contact to loading response to facilitate controlled knee flexion as the foot moves toward the floor. If the patient presents with genu recurvatum in early stance, in some cases reduction of the plantarflexion resistance threshold may permit knee hyperextension before mid-stance (39). In such cases, the plantarflexion resistance threshold may need to be increased and iteration of this adjustment may be necessary to determine the best setting to resist knee hyperextension while permitting ankle plantarflexion as much as possible in early stance (Step 4B: Figure 11). The final setting of the plantarflexion resistance threshold should therefore balance and prioritize these concerns and the clinician must decide on the primary gait deficit to be treated while prioritizing the reduction of other gait deviations.

*Step 5: late stance–phase adjustment (orthosis donned while patient is walking)—approximately 8 min*

The last step of the algorithm involves adjusting the dorsiflexion resistance threshold for the late stance phase of the gait cycle. This adjustment is intended to permit resisted ankle dorsiflexion with knee stability during the second and third rockers (Step 5: Figures 12, 13). The resistance of an AFO to dorsiflexion encourages knee extension after mid-stance and may also help control forward tibial progression during the second rocker (Step 5A: Figure 12). The multi-function articulated AFO will begin resisting dorsiflexion as the ankle attempts to dorsiflex beyond the ankle alignment angle. Resistance to dorsiflexion is essential to compensate for plantarflexor and quadriceps weakness and to encourage full knee extension after mid-stance. However, excessive resistance to dorsiflexion may also result in undesirable knee hyperextension in terminal stance (36). In this step of the algorithm, tibial progression and knee stability are observed from mid-stance through pre-swing.

The timing of heel rise is also observed after mid-stance and at the third rocker (Step 5B: Figure 13). It is generally accepted that the appropriate timing of heel off occurs prior to initial contact of the contralateral foot, but after the contralateral foot swings past the stance foot in the sagittal plane (59). Evidence suggests that the timing of heel off may also be affected by ankle dorsiflexion range of motion (58). Excessive knee flexion or late heel off after mid-stance suggests an insufficient dorsiflexion resistance threshold. If these gait deviations are observed, consider increasing the dorsiflexion resistance threshold. Conversely, the observation of excessive knee hyperextension or early heel off after mid-stance suggests that the dorsiflexion resistance threshold should be decreased.

## 4 Hypothetical case studies

The multi-function Articulated AFO Adjustment Algorithm (Supplementary File 1) is applicable to the orthotic treatment of a broad range of complex neuromotor pathologies. To illustrate the application of this algorithm, two hypothetical clinical cases are presented. These cases are based on the generalized clinical presentation and treatment outcomes of an ensemble of actual patients treated using multi-function articulated AFOs with the adjustment algorithm and by order of a prescribing physician.

### 4.1 Example 1: a patient with myelomeningocele

Imagine a hypothetical patient, a 15-year-old adolescent boy, who presents to clinic with myelomeningocele (MMC). The underlying pathology results in the functional deficit of absent volition of the plantarflexors with other motor function mostly preserved. Because of the plantarflexor deficit, the patient exhibits no push-off in the late stance phase of gait, and walks with persistent knee flexion throughout the stance phase. It is important to keep these deficits in mind when reviewing slow-motion videos during the optimization process. The patient has a history of orthotic treatment using non-articulated plastic AFOs worn with athletic footwear and native outsoles. However, an iatrogenic gait abnormality of excessive knee flexion in the first rocker is observed and persistent knee flexion throughout the stance phase remains unaddressed in the current orthotic design. The goals of orthotic treatment will be to improve the patient's stance phase gait mechanics while minimizing restriction of the ankle to preserve ankle motion in the first and second rockers, to reduce knee flexion in the first rocker of gait, and to achieve full knee extension without knee hyperextension in the late stance phase.

**Figure 14:**
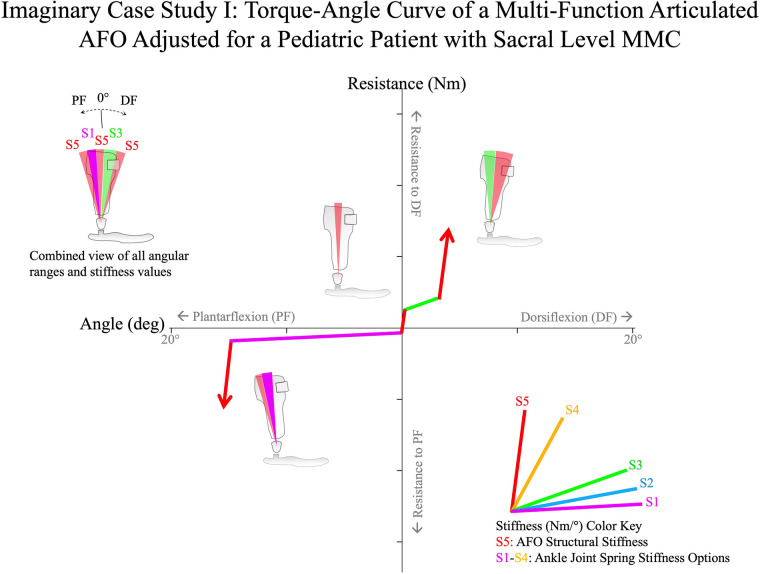
A hypothetical case study of the adjustment algorithm for a 15-year-old adolescent boy with sacral level MMC. With highly active dorsiflexors and absent plantarflexors, the multi-function articulated AFO is adjusted to have low stiffness through 15° of plantarflexion and then encounters the high stiffness of the AFO structure. A brief period of high stiffness is set around neutral (0°) for stance-phase stability, with a moderate spring stiffness into ∼4° of dorsiflexion where the high stiffness of the AFO was encountered to prevent excessive dorsiflexion in the late stance-phase, and improve knee extension in mid-stance. MMC, myelomeningocele.

The patient is molded for bilateral multi-function articulated AFOs (Figure 14). The negative casts are corrected before pouring the positive model to align the sagittal ankle angle of the AFOs. This alignment is intended to promote a slight inclination of the shank when the AFOs are fitted to the patient wearing shoes. The AFOs incorporate features intended to resist the pathologic foot and ankle postural abnormalities.

*Step 1: bench adjustment*

Prior to fitting, the orthoses are bench adjusted. Bench adjustment is performed by adjusting the ankle alignment angle to its neutral setting (at the angle of fabrication that slightly inclines the shank when fit with the shoes) and the resistance threshold of the multi-function articulated AFO ankle joints to their maximum setting, effectively configuring the AFOs as solid ankle-foot orthoses.

*Step 2: static alignment*

The patient is seen for orthotic fitting, and the orthoses and shoes are donned. The fit of the orthoses is evaluated and adjusted to provide comfort and postural support without irritation.

The patient is asked to stand, and the ankle alignment angles are adjusted with the patient in static weight bearing. It is observed that the patient's knees are excessively flexed, therefore the static alignment angle is adjusted toward plantarflexion to slightly recline the shank and provide improved standing balance. Reclining the shank is perceived to shift the visualization of the weight line (TKA line) posteriorly. This patient's knee flexion is observed to be very responsive to the adjustment of ankle angle and is easily optimized during static alignment.

*Step 3: swing phase alignment*

The patient is asked to walk at a comfortable pace while the clinician uses a smart phone to record slow-motion, sagittal plane video. Slow-motion video captures at the high frame rate of 240 frames per second, resulting in a high-resolution video that improves the clarity of stop-motion and scrolled images. The clinician reviews the video, slowly scrolling the image left and right to analyze toe clearance in mid-swing and foot-to-floor angle at initial contact. This assists in identifying the pathologic gait abnormalities. Through the analysis of multiple steps of the patient walking, it appears that the toe clearance is greater than 2 cm in mid-swing, but the foot position at initial contact appears symmetric between the left and right sides. It is also observed that the foot-to-floor angle is excessive and greater than 25°. The ankle alignment settings of the AFOs are adjusted toward plantarflexion to decrease the foot-to-floor angle at initial contact. The walking trial is repeated, and slow-motion smart phone video confirms that the new alignment setting encourages heel contact with decreased dorsiflexion at initial contact and a foot-to-floor angle of about 20°. There does not appear to be any effect of the adjustment on knee extension at terminal swing. The patient's standing balance is again evaluated in static weight bearing. Shank inclination appears slightly reduced, but the knees do not appear hyperextended, and the ankle alignment setting is verified as the best compromise overall that improves standing balance and the patient's sense of stability in ambulation. The final, best-compromise multi-function articulated AFO alignment setting is 0°.

*Step 4: early stance–phase adjustment*

During initial adjustment of the AFO, the plantarflexion and dorsiflexion were locked to simulate a non-articulated AFO with high stiffness. Therefore, it is suspected that the high resistance to plantarflexion might result in the iatrogenic gait abnormality of rapid knee flexion in the first rocker. Slow-motion video confirms this suspicion.

To improve early stance-phase knee kinematics, the Early Stance-Phase Adjustment procedure is performed. Because the patient's dorsiflexion strength is preserved, it was anticipated that a lower stiffness, high compliance spring resisting plantarflexion might be appropriate for the patient. Therefore, a spring of 0.2 Nm/deg stiffness had been installed in the component's plantarflexion-resist channels prior to Bench Adjustment. The plantarflexion resistance threshold of the multi-function articulated AFOs are adjusted to 1 Nm permitting 15° of ankle plantarflexion relative to the ankle alignment angle before encountering the plantarflexion stop.

The patient is again asked to walk, and slow-motion video confirms that the toe clearance in mid-swing and foot-to-floor angle at initial contact remain unchanged after reducing the plantarflexion resistance threshold. The excessive knee flexion in the first rocker is again observed and appears reduced following this adjustment but is still present. Therefore, the plantarflexion resistance threshold is further decreased to 0.6 Nm, increasing the compliance of the AFO in plantarflexion. Video analysis is repeated and reveals that this adjustment appears to significantly reduce the rapid knee flexion in the first rocker of gait. Foot position in swing phase and at initial contact remains unchanged, and ankle plantarflexion is clearly observed from initial contact to foot flat. The patient now ambulates with improved foot position throughout the swing phase and at initial contact and with significantly improved knee kinematics and visible ankle plantarflexion during the first rocker.

*Step 5: late stance–phase adjustment*

Having remediated the iatrogenic gait abnormality of rapid knee flexion in the first rocker, Late Stance Phase Adjustment is performed. A stated goal of orthotic treatment was encouraging full knee extension in late stance phase. The orthosis had been bench adjusted for high structural resistance to dorsiflexion and this setting has not yet been changed. Therefore, the orthosis had been configured to block ankle dorsiflexion beyond the ankle alignment angle that occurs at mid-stance. While achieving full knee extension was a stated goal, knee hyperextension is observed and considered an iatrogenic gait abnormality; therefore, the resistance threshold to ankle dorsiflexion must be decreased.

A 0.3 Nm/deg stiffness spring had initially been installed in the component's dorsiflexion-resist channel to provide assertive resistance to ankle dorsiflexion over a shorter range of motion substituting for the absent plantarflexors. The dorsiflexion resistance threshold is changed to 0.6 Nm, which permits a maximum of 16° of dorsiflexion range of motion relative to the ankle alignment angle before encountering the dorsiflexion stop. The patient is asked to walk, and slow-motion video confirms that the knee hyperextension has decreased after mid-stance, but repeated observations reveal that at this dorsiflexion resistance threshold, full knee extension is achieved only intermittently. Therefore, the dorsiflexion resistance threshold is increased to 1.4 Nm and the patient is again asked to walk. With this adjustment, the video confirms that the patient achieves reliable, full knee extension with smooth tibial progression through mid-stance without knee hyperextension or early heel rise.

After completion of the algorithm, the patient's gait pattern is comprehensively reviewed to determine whether there are additional opportunities for improvement through iteration of multi-function articulated AFO component settings.

### 4.2 Example 2: a patient with Charcot–Marie–Tooth disease

Imagine a hypothetical 76-year-old elderly man with a history of Charcot–Marie–Tooth (CMT) presents to the clinic with a plantarflexion contracture with maximum dorsiflexion of 0° and quadriceps weakness. The patient's ambulatory function is impaired with several pathologic gait abnormalities including poor foot clearance in swing phase, steppage gait, short step length, and slow walking. Without use of an assistive device, the patient walks with an anterior trunk lean and instability. The patient's chief complaint is decreased activity level and an increased number of falls.

The primary goal of orthotic treatment is to provide support for the quadriceps and decrease the risk of falls. Secondary goals are to improve standing balance in static weight bearing, and to improve toe clearance in swing phase while minimizing restriction of the ankle to preserve ankle motion in the first and second rockers.

**Figure 15:**
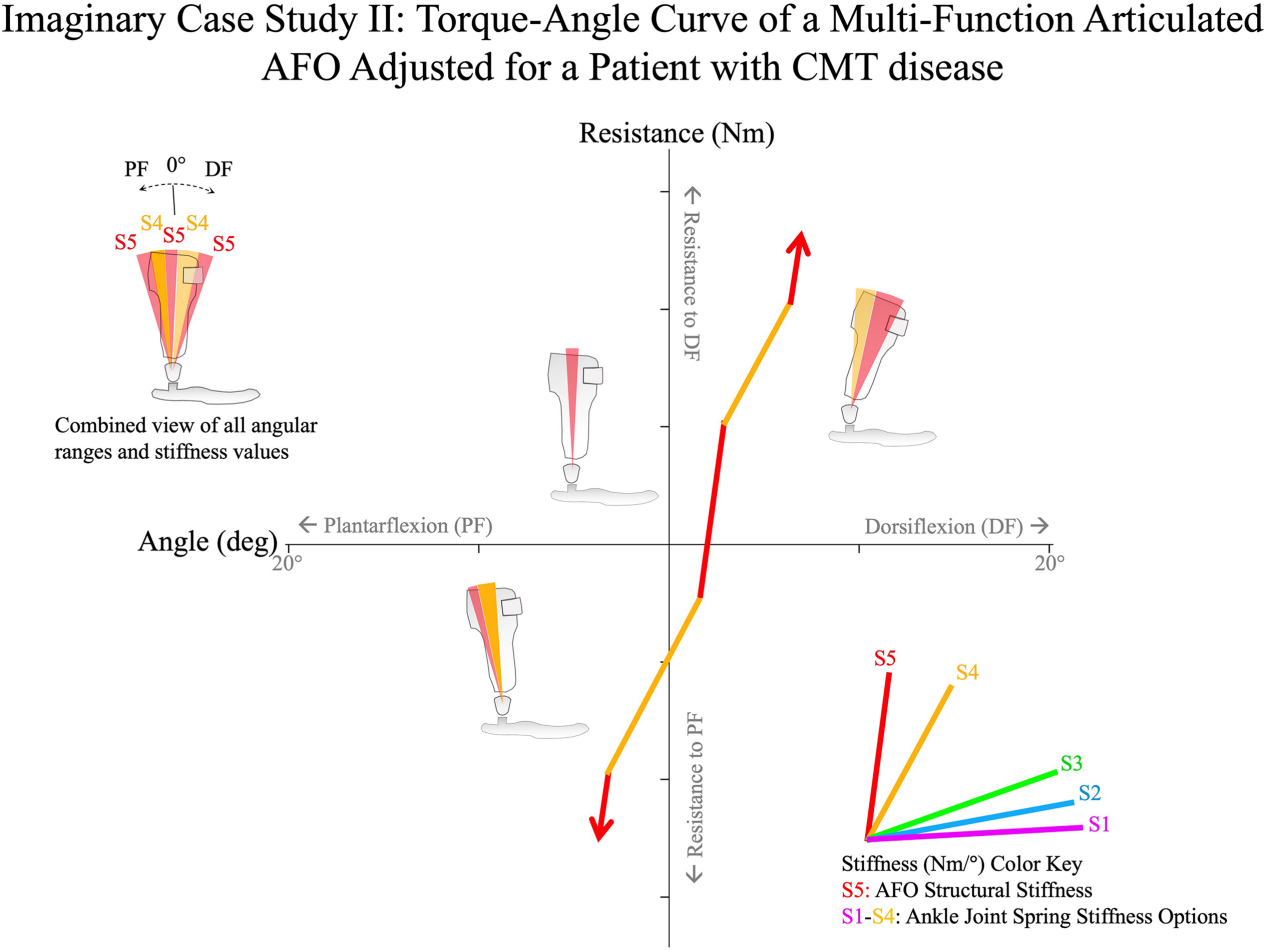
A hypothetical case study of the adjustment algorithm for a 76-year-old elderly man with CMT disease. The patient's pathologic gait deviations are the result of bilateral plantarflexion contractures at 0° and quadriceps weakness. After optimization of the ankle alignment angle for balance in static weight bearing, the multi-function articulated AFO is adjusted using the algorithm to allow 8° of resisted plantarflexion against stiff springs. The resistance threshold to dorsiflexion is adjusted to permit the second rocker against high resistance springs to stabilize the knees before encountering the high structural stiffness of the AFO at 5° dorsiflexion. CMT, Charcot-Marie-Tooth.

The patient is molded for fabrication of bilateral multi-function articulated AFOs (Figure 15). Prior to fabrication, the negative casts are corrected to neutral (0°) dorsiflexion, which would facilitate approximately 5° of shank inclination when fitted with shoes. This ankle angle is the patient's maximum passive dorsiflexion range of motion.

*Step 1: bench adjustment*

Prior to fitting, the orthoses are bench adjusted. Bench adjustment is performed by adjusting the ankle alignment angle to its neutral setting (at the angle of fabrication) and the resistance threshold of the multi-function articulated AFO ankle joints to their maximum resistance setting, effectively configuring the AFOs as solid ankle-foot orthoses.

*Step 2: static alignment*

The patient is seen for orthotic fitting and the orthoses and shoes are donned. The fit of the orthoses is evaluated and adjusted. After achieving the appropriate fit to provide comfort and postural support without irritation, the patient's AFOs are optimized using the evidence-guided algorithm.

The patient is asked to stand in the orthoses and the ankle alignment angles are adjusted with the patient in static weight bearing. Because the patient's ankle dorsiflexion range of motion is limited, heel lift insoles are added to his shoes plantar to the orthoses to accommodate the contractures and maintain the position of the ankles within their passive range of motion. This facilitates optimization of shank inclination while avoiding alignment of the ankle angle at the patient's end of anatomic dorsiflexion range of motion. The shank inclination is evaluated, and patient feedback is solicited regarding his sense of stability in quiet standing. The final ankle alignment setting is 2° dorsiflexion. This static alignment results in the patient standing in slight knee flexion. Because the orthoses present high resistance to dorsiflexion (with dorsiflexion blocked at bench adjustment), the patient has the sense of improved standing balance, which is objectively observed in a more relaxed and erect trunk and arm position. When solicited for feedback, the patient expresses a feeling of improved stability and comfort.

*Step 3: swing-phase alignment*

The patient is asked to walk at a comfortable pace. A smart phone is used to record slow-motion video to assist in identifying pathologic gait abnormalities.

Through this analysis it is observed that when walking, the patient has improved toe clearance and foot position at initial contact with the initial bench adjustment, although foot position at initial contact and step length appear slightly asymmetrical between the left and right sides. The ankle alignment settings of the AFOs are adjusted to improve symmetry while ensuring that the foot-to-floor angle is maintained at approximately 10° at initial contact. Following this adjustment, the patient expresses the feeling of greater stability while walking and this is reflected by the observed decrease in anterior lean and reduced trunk sway during gait. The patient's sense of standing balance is again evaluated in static weight bearing and the ankle alignment setting is verified as the best compromise that overall provides the best standing balance and sense of stability while the patient is walking.

*Step 4: early stance–phase adjustment*

After the pathologic gait abnormalities of foot clearance in mid-swing, knee extension at terminal swing, foot position at initial contact, and step length symmetry in early stance have been remediated with static- and swing-phase alignment, it is anticipated that the high resistance to plantarflexion of the multi-function articulated AFOs might result in the iatrogenic gait abnormality of rapid knee flexion in the first rocker. This is confirmed by observation. To improve early stance-phase kinematics, the Early Stance-Phase Adjustment procedure is performed.

It was anticipated that a high stiffness spring resisting plantarflexion was appropriate for the patient, due to the patient's weight and the nature of their biomechanical deficits; therefore, a spring of 1.5 Nm/deg stiffness was installed in the component's plantarflexion-resist channels prior to Bench Adjustment.

The plantarflexion resistance thresholds of the multi-function articulated AFOs are adjusted to 4.3 Nm facilitating 5° of plantarflexion range of motion relative to the ankle alignment angle before encountering the plantarflexion motion stop. However, it is observed that knee flexion is still exaggerated in the first rocker from initial contact to loading response at this plantarflexion resistance threshold setting. Therefore, the plantarflexion resistance threshold is further reduced to 1 Nm. The evaluation is repeated and this change in component settings appears to result in improved knee stability in the first rocker with controlled knee flexion through early stance, while maintaining the position of the foot from swing to initial contact.

*Step 5: late stance–phase adjustment*

Having optimized knee kinematics in the first rocker, attention is lastly focused on Late Stance–Phase kinematics. Tibial progression through mid-stance and knee kinematics at terminal stance are evaluated using slow-motion video.

It was anticipated that a high stiffness spring resisting dorsiflexion would be appropriate for the patient, due to the patient's weight and the weak quadriceps; therefore, a spring of 1.5 Nm/deg stiffness had been installed in the component's dorsiflexion-resist channels prior to Bench Adjustment. With the AFOs still adjusted to block dorsiflexion, repeated observations using slow-motion video of the patient walking confirm that while knee stability appears improved and gait speed is higher, tibial progression is interrupted in the second rocker near the static alignment angle.

Therefore, the resistance threshold to dorsiflexion is decreased to 5 Nm to allow resisted ankle dorsiflexion past the ankle alignment angle after mid-stance, facilitating 4° of resisted dorsiflexion range of motion beyond the ankle alignment angle. Resisted dorsiflexion is intended to support the quadriceps and keep the knee more extended through mid-stance while improving tibial progression in the second rocker until the structural stiffness of the orthosis is encountered at the end of dorsiflexion range of motion.

After completion of the algorithm, the patient's gait pattern is comprehensively reviewed to determine whether there are additional opportunities for improvement through iteration of multi-function articulated AFO component settings.

## 5 Discussion and limitations of the algorithm

The overarching goal of this AFO adjustment algorithm is to mitigate pathologic gait deviations while minimizing restriction of ankle motion throughout the gait cycle. It is assumed that the mitigation of pathologic gait deviations will improve overall patient function and the least possible ankle restriction will facilitate the most beneficial therapeutic outcome. However, the evidence is limited to the biomechanical principles of the steps for optimizing multi-function articulated AFOs rather than the efficacy of orthotic treatment due to the lack of research in this area.

The method was developed to assist in the optimization of multi-function articulated AFOs in the orthotic treatment of pathologic gait secondary to stroke, CP, traumatic brain injury (TBI), MMC, multiple sclerosis (MS), CMT disease, and other neuromotor pathologies. By adopting the modest ambition of developing a preliminary adjustment algorithm focused on the reduction of pathologic gait deviations when compared to normal gait, the algorithm is intended to serve as a preliminary guideline for the adjustment of AFO mechanical characteristics to streamline the process of AFO adjustment and improve the consistency of the clinician's approach in reducing the pathologic gait deviations that may result from a broad range of underlying pathologies.

Evidence from published research that supported the development of the algorithm suggests that the method could potentially be used to systematically reduce pathologic gait deviations, thereby improving gait, daily activities, and the quality of life for patients with a broad range of underlying pathologies. The focal influence of specific mechanical characteristics of multi-function articulated AFOs on certain kinematic variables, and the reliability of observational gait analysis augmented by repeated observations of specific gait events using slow-motion video, were established and lay the foundation for the method (8, 11, 26, 31, 36, 39, 40, 44–49, 53, 54). However, some observations employed by the algorithm are less well supported including knee extension at terminal swing and timing of heel rise at late stance. Additional research is required to validate the utility of these clinical observations for orthotic adjustment. There is also insufficient evidence to support the efficacy of AFOs in general in the treatment of patients with pathologic gait abnormalities (60, 61). Multi-site studies using an ensemble of metrics including patient activity level, kinematic measurements, and validated performance-based and patient-reported outcome measures to determine patient satisfaction and quality of life could address these limitations.

Identification of pathologic gait deviations plays an important role in our proposed algorithm. Although there have been significant advances in motion analysis technology, a cost-effective and clinically viable means to quickly and accurately assess gait performance remains unrealized. In clinical practice, orthotists rely heavily on observational gait analysis to assess the impact of orthotic treatment. However, evidence also suggests that the reliability of observational gait analysis may be influenced by the clinician's skill level and personal experience (44, 46). There is also evidence suggesting that this reliability may be improved by focusing the clinician's observations on specific gait events, with repetitive trials using slow-motion video (45, 47–49). A thorough validation of the algorithm is necessary in future studies with a large sample size.

The published research does not support application of a single AFO stiffness for treating the complex gait pathologies observed (39, 62). Therefore, our case examples illustrate how the method could be applied to adjust the mechanical characteristics including stiffness and the resistance threshold of an AFO to the unique needs of the individual patient to achieve the best possible results. This method was developed to be effectively implemented in the clinical setting by orthotists familiar with the basic techniques of customary orthotic practice. Real-world functional gait data that demonstrate the efficacy of orthotic treatment and AFOs optimized using the method are not available; however, this limitation could be overcome by conducting large-scale clinical trials with comprehensive evaluations of a variety of patient populations (63).

Future applications of the algorithm (Supplementary File 1) could involve developing a structured methodology for orthotic clinical care. This could inform research by standardizing orthotic practice, therefore facilitating the isolation of variables essential to experimental design. Such research could focus on efficacy, potentially leading to advancements in care delivery, orthotic design, and ultimately improving patient outcomes.

## Data Availability

The original contributions presented in the study are included in the article/Supplementary Material, further inquiries can be directed to the corresponding author.

## Supplementary material

Supplementary File 1 Summary of the adjustment process of multi-function articulated ankle-foot orthoses.
