# Supplementary material for: A proposed evidence-guided algorithm for the adjustment and optimization of multi-function articulated ankle-foot orthoses in the clinical setting
Source: Front Rehabil Sci. 2024 Jul 24;5:1353303. doi: 10.3389/fresc.2024.1353303 (PMC11307126; doi:10.3389/fresc.2024.1353303)
Supplement: Supplementary File 1 — Summary of the adjustment process of multi-function articulated ankle-foot orthoses. [file Datasheet1.pdf]

A Proposed Evidence-Guided Algorithm for the Adjustment and Optimization of Multi-Function Articulated Ankle-Foot Orthoses in the Clinical Setting

LeCursi N, Janka B, Gao F, Orendurff M, He Y, Kobayashi T

STEP 1 : Bench Adjustment

Align ankle to incline shank. Adjust the plantarflexion and dorsiflexion resistance thresholds to maximum (AFO solid with ROM = 0°)

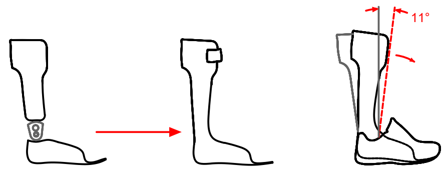

AFO ADJUSTMENT
Adjust the PF and DF resistance thresholds to their maximum levels

AFO ADJUSTMENT
Align ankle joint to incline shank near 11° SVA

STEP 2: Static Alignment

Adjust ankle alignment with AFO solid (ROM = 0°)

Gait Deviation
Shank reclined with knee extended and weight line toward heel

AFO Adjustment
Dorsiflex alignment to incline shank and shift weight line toward mid foot

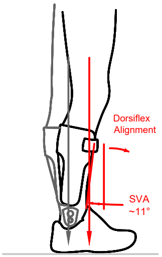

Gait Deviation
Shank inclined with knee flexed and weight line toward forefoot

AFO Adjustment
Plantarflex alignment to recline shank and shift weight line toward mid foot

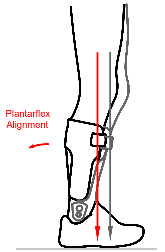

Quiet Standing

STEP 3: Swing Phase Alignment (Iterate with STEP 2)

Iteratively adjust ankle alignment with AFO solid (ROM = 0°)

Gait Deviation
Not enough toe clearance in mid swing

AFO Adjustment
Dorsiflex alignment to increase toe clearance

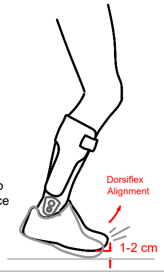

Mid Swing

Gait Deviation
Not enough knee extension at terminal swing with or without shortened step length

AFO Adjustment
Plantarflex alignment to increase knee extension and equalize step lengths between left to right

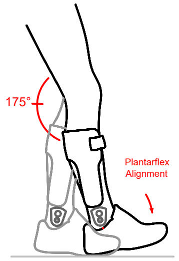

Terminal Swing

Gait Deviation
Foot-to-floor angle too low at initial contact

AFO Adjustment
Dorsiflex alignment. Ensure symmetrical foot-to-floor angles for both the left and right feet

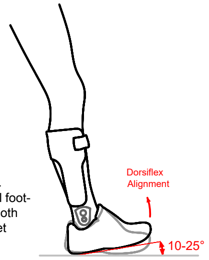

Gait Deviation
Foot-to-floor angle too high at initial contact

AFO Adjustment
Plantarflex Alignment. Ensure symmetrical foot-to-floor angles for both the left and right feet

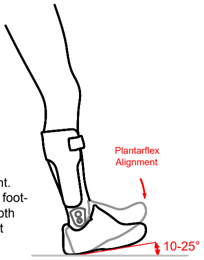

Initial Contact

STEP 4: Early Stance Phase Adjustment

Adjust the plantarflexion resistance threshold (PF Rt) to optimize knee joint kinematics. (DF ROM = 0°)

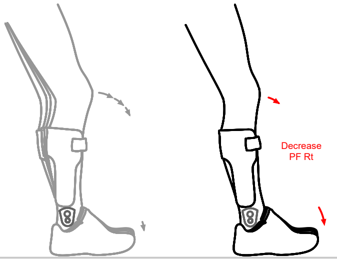

Gait Deviation
Rapid knee flexion in the 1st rocker with limited ankle plantarflexion

AFO Adjustment
Decrease the plantarflexion resistance threshold (PF Rt) to reduce rapid knee flexion in the 1st rocker

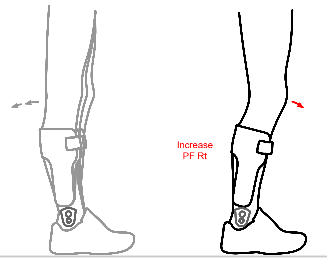

Gait Deviation
Knee hyperextension before midstance

AFO Adjustment
Increase the plantarflexion resistance threshold (PF Rt) to reduce knee hyperextension before midstance

1st Rocker

STEP 5: Late Stance Phase Adjustment

Adjust the dorsiflexion resistance threshold (DF Rt) to optimize knee joint kinematics and timing of heel rise.

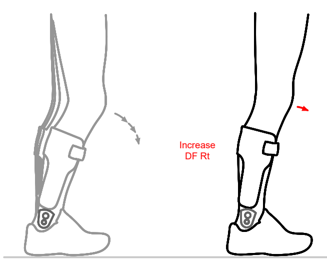

Gait Deviation
Excessive knee flexion after midstance

AFO Adjustment
Increase dorsiflexion resistance threshold (DF Rt) to reduce knee flexion after midstance

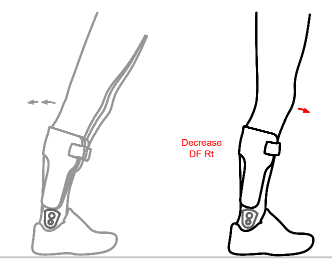

Gait Deviation
Knee hyperextension after midstance

AFO Adjustment
Decrease dorsiflexion resistance threshold (DF Rt) to reduce knee hyperextension after midstance

2nd Rocker

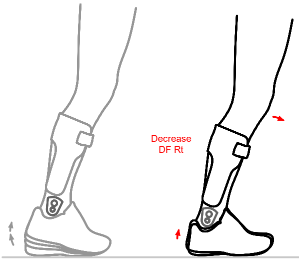

Gait Deviation
Early heel rise with or without knee hyperextension

AFO Adjustment
Decrease the dorsiflexion resistance threshold (DF Rt) to delay heel rise

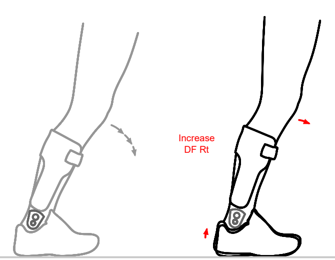

Gait Deviation
Late heel rise with or without knee flexion

AFO Adjustment
Increase the dorsiflexion resistance threshold (DF Rt) to accelerate heel rise

3rd Rocker
